# Human placental transcriptome shows sexually dimorphic gene expression and responsiveness to maternal dietary n-3 long-chain polyunsaturated fatty acid intervention during pregnancy

**DOI:** 10.1186/1471-2164-15-941

**Published:** 2014-10-27

**Authors:** Eva-Maria Sedlmeier, Stefanie Brunner, Daniela Much, Philipp Pagel, Susanne E Ulbrich, Heinrich HD Meyer, Ulrike Amann-Gassner, Hans Hauner, Bernhard L Bader

**Affiliations:** ZIEL-PhD Graduate School ‘Epigenetics, Imprinting and Nutrition’, Research Center for Nutrition and Food Sciences (ZIEL), Technische Universität München, Freising-Weihenstephan, Germany; Else Kröner-Fresenius-Center for Nutritional Medicine, Technische Universität München, Freising-Weihenstephan, Germany; Else Kröner-Fresenius-Center for Nutritional Medicine, Klinikum rechts der Isar, Uptown München Campus D, Munich, Germany; Chair in Genome oriented Bioinformatics, Technische Universität München, Freising-Weihenstephan, Germany; Physiology Unit, Research Center for Nutrition and Food Sciences (ZIEL), Technische Universität München, Freising-Weihenstephan, Germany; Clinical Nutritional Medicine Unit, Research Center for Nutrition and Food Sciences (ZIEL), Technische Universität München, Gregor-Mendel-Strasse 2, 85350 Freising-Weihenstephan, Germany

**Keywords:** Human placenta, Transcriptome, Sexual dimorphism, N-3 LCPUFA, Pregnancy, Fetal programming, Sex steroid hormones

## Abstract

**Background:**

Previously we have examined the effect of maternal dietary n-3 long-chain polyunsaturated fatty acid (LCPUFA) supplementation during pregnancy on offspring fat mass. Considering the involvement of the placenta in fetal programming, we aimed to analyze the sex-specific gene expression in human term placenta and its response to the n-3 LCPUFA intervention, as well as their correlations to offspring adiposity.

**Results:**

Placental gene expression was assessed in a control and n-3 LCPUFA intervention group by DNA microarrays, biological pathway analyses and RT-qPCR validation. Expression data were correlated with sex steroid hormone levels in placenta and cord plasma, and offspring anthropometric data. Transcriptome data revealed sexually dimorphic gene expression in control placentas *per se*, whereas in intervention placentas sex-specific expression changed, and more n-3 LCPUFA-regulated genes were found in female than male placentas. Sexually dimorphic gene expression and n-3 LCPUFA-responsive genes were enriched in the pathway for cell cycle and its associated modulator pathways. Significant mRNA expression changes for *CDK6*, *PCNA*, and *TGFB1* were confirmed by RT-qPCR. *CDK6* and *PCNA* mRNA levels correlated with offspring birth weight and birth weight percentiles. Significantly reduced placental estradiol-17β/testosterone ratio upon intervention found in female offspring correlated with mRNA levels for the 'Wnt signaling' genes *DVL1* and *LRP6*.

**Conclusions:**

Overall, human placentas show sexually dimorphic gene expression and responsiveness to maternal n-3 LCPUFA intervention during pregnancy with more pronounced effects in female placentas. The absence of correlations of analyzed placental gene expression with offspring adipose tissue growth in the first year is not mutually exclusive with programming effects, which may manifest later in life, or in other physiological processes.

**Electronic supplementary material:**

The online version of this article (doi:10.1186/1471-2164-15-941) contains supplementary material, which is available to authorized users.

## Background

The concept of ‘developmental origins of health and disease’ (DOHaD) hypothesizes that intrauterine nutritional imbalances cause an early adverse growth and can trigger adaptation mechanisms predisposing offspring to complex disease in later life, such as obesity, metabolic, and cardiovascular disease [1]. The prevalence of obesity in US children and adolescents was 16.9% in 2009–2010 [2], and a German survey showed only slightly lower rates [3]. As a strategy for primary obesity prevention, Ailhaud and Guesnet proposed to reduce the n-6/n-3 fatty acid ratio of the maternal diet during pregnancy [4]. This was based on their observation that offspring of mice fed a high-fat diet with low n-6/n-3 fatty acid ratio before mating and during the pregnancy/lactation period had lower body weight from weaning until adulthood compared to offspring of mice fed a high-fat diet with a high n-6/n-3 fatty acid ratio [4, 5]. Moreover, for the n-6 long-chain polyunsaturated fatty acid (LCPUFA) arachidonic acid it was shown to stimulate adipocyte differentiation and maturation, whereas n-3 LCPUFAs eicosapentaenoic acid (EPA) and docosahexaenoic acid (DHA) were counteractive [5]. As a proof-of-concept, we previously performed a randomized, controlled human intervention trial, the INFAT (**I**mpact of **N**utritional **F**atty Acids during Pregnancy and Lactation on Early Human **A**dipose **T**issue Development) study, and addressed as primary endpoint whether a reduced n-6/n-3 LCPUFA ratio in maternal nutrition during pregnancy and lactation might represent a strategy to reduce offspring adipose tissue growth [6]. In the INFAT study also placentas from offspring were collected for further research exploring the impact of the n-3 LCPUFA intervention on placental gene expression, as described in the present work.

The placenta is suggested to play a key role in mediating fetal metabolic programming of the offspring *in utero*, because as central interface between mother and fetus the placenta senses and adapts to environmental stimuli, especially nutritional stimuli, with alterations in nutrient transport and hormone production (reviewed in [7, 8]). For human and mouse models, it has been reported that male and female fetuses differentially respond to *in utero* environmental stimuli [9, 10]. With regard to adipose tissue growth and distribution, differences between male and female are already apparent at birth [11]. Sex-specific differences have also been described for the metabolism and status of LCPUFAs in human subjects [12]. For instance, higher n-3 LCPUFA tissue concentrations, especially for DHA, were found in female than in male adults associated with a higher synthesis rate of n-3 LCPUFAs from their precursor alpha-linolenic acid in females. Furthermore, Ryan *et al*. [13] found that DHA-supplementation of formula in pre-term children sex-specifically decreased the gain of body weight, body length, and head circumference. In addition, they observed a lower fat-free mass in 6 months old boys, but not in 6 months old girls.

LCPUFAs and their derivatives can exert their biological effects by modulating signal transduction via membrane receptors or membrane composition, or serving as ligands of transcription factors, such as PPARα-γ [14]. For human placenta, Sood et al. reported gene expression differences between human male and female placentas [15]. The placenta tissue carrying the fetal genome and sex appears as promising candidate to be involved in mediating sex-specific functions and programming effects on the fetus with sustainable impact [9, 10, 16]. In general, the underlying mechanisms of sexually dimorphic gene expression are not well understood. It has been shown that genes located on sex chromosomes contribute to differential gene expression between male and female somatic tissues, and sex steroid hormones are thought to provide the initiation of sexual differentiation [10, 17–19].

Considering the sex-specific differences in the metabolism and status of LCPUFAs in human subjects, the roles of n-6 and n-3 LCPUFAs in the regulation of gene expression and fetal and postnatal development, and that maternal-derived n-6 and n-3 LCPUFAs are transported via the placenta to the fetus [20, 21], we aimed in the present study to explore whether the placental transcriptomes between female and male offspring are different *per se*, and if maternal n-3 LCPUFA intervention during pregnancy has a sex-specific impact on the placental transcriptome. Furthermore, we investigated if observed diffential gene expression is related to sex steroid hormone levels in placenta and umbilical cord blood plasma, and may have a programming effect on offspring body composition. To answer these questions, we analyzed the gene expression in the placentas of offspring from defined control (Con) and n-3 LCPUFA intervention (N3) subpopulations of the INFAT study by transcriptome and pathway analyses, followed by quantitative real-time polymerase chain reaction (RT-qPCR) validation. We measured placental and umbilical cord plasma sex steroid hormone levels, and analyzed the gene and protein expression for aromatase (*CYP19A1*). Correlation analyses were performed between the mRNA levels of specific sexually dimorphic expressed genes and sex steroid hormone levels, as well as offspring anthropometric data. To evaluate the relation between placental sexually dimorphic gene expression and sex steroid hormone levels on genomic level, the DNA sequences of identified regulated target genes were searched for putative hormone-responsive elements of the respective hormone receptors.

## Methods

### Ethics statement and subpopulations of the INFAT study

The present study on human placental gene expression is a substudy of our previously published randomized, controlled human intervention trial, the INFAT (**I**mpact of **N**utritional **F**atty Acids during Pregnancy and Lactation on early Human **A**dipose **T**issue Development) study [6]. The data of the INFAT study addressed as primary endpoint whether a reduced n-6/n-3 LCPUFA ratio in maternal nutrition during pregnancy and lactation might represent a strategy to reduce offspring adipose tissue growth [6]. The study protocol was in accordance with the rules of the International Conference on Harmonization Good Clinical Practice guidelines (valid from 17 January 1997), the last revision of the declaration of Helsinki (October 2000; Edinburgh, United Kingdom) and applicable to local regulatory requirements and laws. The study protocol was approved by the ethical committee of the Technische Universität München (1479/06/2006/2/21) and registered at clinicaltrials.gov as NCT00362089. The INFAT study was an open-label, monocenter, prospective, randomized, controlled dietary intervention trial in a 2-arm parallel group design, as described in detail by Hauner et al. [22]. All participants gave written consent prior to their inclusion in the study. All human materials, including term placentas and cord blood plasma, were obtained from participants of the INFAT study by written informed consent according to the consent regulation [6, 22].

In brief, in the INFAT study 208 women of Western European descent with a BMI between 18 and 30 without high-risk pregnancies or prior n-3 fatty acid supplementation were included before the 15^th^ week of gestation and randomly assigned to a control (Con) or n-3 LCPUFA intervention group (N3). Both groups were advised for a healthy diet during pregnancy. The women of the intervention group were additionally advised to decrease their dietary n-6/n-3 LCPUFA ratio to about 3:1 by a daily n-3 LCPUFA supplementation (1,020 mg DHA and 180 mg EPA) and reduction of arachidonic acid intake to 50–90 mg per day. Healthy term-born offspring (between 37^th^ and 42^nd^ week of gestation) were included for further analyses. Mode of delivery, sex of the newborn, birth weight and length, gestational age, and birth weight percentiles were recorded from maternal obstetric records. Anthropometric measurements (weight, skin folds) and abdominal ultrasonography were conducted at 3–5 days, 6 weeks and one year *post partum*, and birth weight / birth length ratio, birth weight / placental weight ratio, sum of four skin-fold thicknesses and subcutaneous / preperitoneal fat ratio were calculated as reported previously [6]. Excellent compliance was already demonstrated for the entire INFAT study population (n = 208), including also all women of the present INFAT substudy. Moreover, this compliance was also reflected by LCPUFA biomarker for maternal red blood cells (RBC) and offspring cord RBC of the subpopulations, comprising INFAT participants and their offspring from the control (Con) and n-3 LC-PUFA intervention (N3) group and used in the present study for transcriptome analysis (n = 16) and validation experiments (n = 41) as shown in Additional file 1: Table S1 and S2. The data for the individual subjects are derived from already published data of the whole INFAT subpopulation [6, 23].

The selection of the analyzed INFAT study subpopulations for this study was determined by minimizing the influences of birth mode and consideration of the use of analgesics or anesthetics on placental gene expression [24–27]. Therefore, all analyzed placentas were derived from offspring of mothers with healthy pregnancies excluding mothers and offspring with pregnancy complications. These offspring were born by vaginal delivery and considered as appropriate for gestational age (10^th^ - 90^th^ percentile of birth weight). For subsequent analyses, data from study participants and their offspring, including placenta, were divided in control group (Con, n = 20) and n-3 LCPUFA intervention group (N3, n = 21). To identify sex-specific effects, data were stratified for offspring sex resulting in additional six groups: group 1: female offspring of Con (Con-F, n = 11); group 2: male offspring of Con (Con-M, n = 9); group 3: female offspring of N3 (N3-F, n = 10); group 4: male offspring of N3 (N3-M, n = 11), group 5: female offspring of Con and N3 together (Con/N3-F, n = 21); group 6: male offspring of Con and N3 together (Con/N3-M, n = 20). The main clinical characteristics of subpopulation mothers and offspring are presented in Additional file 1: Table S2.

### Placenta and cord blood sampling

Having obtained written informed consent, placenta was weighed and placental samples were dissected from each of the four quadrants with the same distance to the placenta center according to a standardized sampling protocol. Regions with large vessels or calcifications were avoided for sampling. To obtain the chorionic villous portion of the placenta, the maternal basal and fetal chorionic plate of each placenta sample were removed. Placenta samples were immediately snap-frozen. Cord blood was collected into EDTA tubes and centrifuged at 4°C with 2,000 g for 10 min. Cord plasma and placenta samples were stored at −80°C until further processing.

### Extraction of total RNA

For each placenta, tissue samples from the four quadrants were homogenized in TRIzol® Reagent (Invitrogen, Darmstadt, Germany) and pooled in equal parts for total RNA extraction by midi RNeasy Kit (Qiagen, Hilden, Germany). Total RNA was quantified with NanoDrop™ 1000 Spectrophotometer (Peqlab, Erlangen, Germany). RNA integrity number (RIN) was analyzed by the Bioanalyzer 2100 (Agilent Technologies, Böblingen, Germany).

### DNA microarray analysis

For transcriptome analysis, the influences of birth mode and the use of analgesics or anesthetics on placental gene expression [24–27] were minimized by using only placentas from offspring that were obtained by vaginal delivery, were appropriate for gestational age newborns (10^th^ - 90^th^ percentile of birth weight), and where no cervical ripening agents (mostly n-6 LCPUFA derivatives), analgesics or anaesthetics were administered during labor of healthy pregnancies. Hence, experiments and data analyses were performed using female (Con-F, n = 4) and male (Con-M, n = 3) placentas from the control group, and female (N3-F, n = 4) and male (N3-M, n = 5) placentas from the n-3 LCPUFA intervention group. The main clinical characteristics of mothers and offspring of the subpopulation for DNA microarray analysis are presented in Additional file 1: Table S1.

Total RNAs with a mean RIN of 6.8 ± 0.3 (SD) were hybridized to Affymetrix Custom Array - NuGO_Hs1a520180 array, containing 17699 genes [28]. All steps and controls of DNA microarray processing were performed according to the manufacturer’s protocol (Affymetrix Inc., Santa Clara, CA, USA). RNA from each individual placenta was applied to individual DNA microarrays which were then processed altogether. MADMAX (Management and Analysis Database for Multi-platform microArray eXperiments) was used for quality control, normalization [*gc Robust Multichip Average* (slow)], annotation by custom CDF-file 13.0.0, and statistical analysis [29]. For Principal Component Analysis (PCA), normalized MADMAX DNA microarray data were used and analyzed with the "prcomp" function of the R statistical software. In MADMAX, fold changes and *P*-values were calculated in moderated t-tests using the R Bioconductor LIMMA package.

For the identification of significantly regulated genes in the placenta considering the effects of n-3 LCPUFA intervention and sex, DNA microarray data analyzed by MADMAX program were further processed in six different comparisons as described in detail in the Results section. Fold changes (FC) of ≤ −1.5 and ≥ +1.5 (*P* < 0.05) were considered as significantly differential gene expression.

DNA microarray data have been curated, accepted and deposited in NCBI’s Gene Expression Omnibus [30] and are accessible under GEO Series accession number GSE53291 (http://www.ncbi.nlm.nih.gov/geo/query/acc.cgi?acc=GSE53291).

### Pathway analysis

The metabolic pathways from *wikipathways*_*Homo*_*sapiens*_*Curation*-*AnalysisCollection*_*gpml* (downloaded 2010-11-04) and *Hsa*-*KEGG*_*20100914* were combined and loaded into *PathVisio 3.1.3*, together with the gene database *Hs*_*derby*_*20100601.bridge* [31]. Positive Z-scores > 0 indicated pathways which contain significantly regulated genes from the DNA microarray data sets in an overrepresentative manner [32].

To indicate the mean direction of gene expression changes for all genes expressed in the respective pathways, the mean centroid value analyses were performed. For this analysis, all log2 intensity data measured by DNA microarray for the genes assigned to the pathway were normalized to a mean of zero and a variance of one per gene across all placentas of the respective comparison (e.g. only control placentas were used for the comparison Con-M vs. Con-F). A mean centroid value for each placenta over all genes per pathway was calculated [33]. From this mean centroid value per placenta a further mean for each analysis group (Con-M, Con-F, Con-M + F, N3-M, N3-F, N3-M + F) in the five comparisons was used to indicate the mean direction of gene expression changes for the pathways.

### Gene expression analysis

RT-qPCR was conducted with 10 ng of total RNA and the QuantiTect SYBR Green RT-PCR Kit (Qiagen, Hilden, Germany) in combination with self-designed intron-spanning primer pairs or validated quantitect primer assays (Qiagen, Hilden, Germany; see Additional file 1: Table S3). The mean RIN of the analyzed RNAs was 6.8 ± 0.4 (SD). The cycling conditions were: 1 cycle at 50°C for 30 min, 1 cycle at 95°C for 15 min, 40 cycles at 95°C for 15 sec/60°C (or as stated in Additional file 1: Table S3) for 30 sec/72°C for 30 sec, and a terminal melting curve. Reactions were controlled by ‘no RT’ and ‘no template’ controls. Melt curve analysis showed, that all RT-qPCR reactions for the analyzed samples in each primer pair were free of primer dimers. Efficiency of primer pairs was assessed by LinRegPCR [34]. Efficiency of used primer pairs was 1.866 ± 0.037 (SD) and therefore, no efficiency correction was applied for calculation of relative gene expression levels. Relative gene expression levels were calculated by the 2^(−ΔΔCt) method [35], including normalization to *ACTB*, *B2M*, *POLR2A*, and *TOP1* as reference genes which were selected from literature for their application as reference genes in placenta tissue [36, 37]. In our study, the suitability of these reference genes was confirmed, since no significant differences between the analyzed groups were observed after analyzing the Cq mean for all four genes together and by bestkeeper [38].

### Sex steroid hormone analysis in placental tissue and cord plasma

Concentrations of the sex steroid hormones progesterone, testosterone (T), estradiol-17β (E2) and total estrogen in cord plasma and placenta samples were determined using enzyme immunoassays according to Prakash et al. [39], Blottner et al. [40] and Meyer et al. [41], respectively. For the progesterone ELISA, a primary antibody directed against progesterone-7α-carboxyethylthioether (kindly donated by Frank Weber, Clinic for Ruminants, Ludwig-Maximilians Universität München, Germany) was used. Plasma aliquots from cord blood (50 μl) were extracted with 30% tertiary butylmethylether/70% petroleum ether (v/v). After freezing at −60°C over night, the supernatants were decanted, dried, diluted in assay buffer, and subjected to the respective ELISA. To determine the total amount of conjugated estradiol-17β and total estrogen, the water residue after extraction was hydrolyzed with β-glucuronidase / arylsulfatase (from Helix pomatia; Merck, Grafing, Germany) at 37°C for 2 h according to Meyer et al. [42] and subjected to a second alcohol extraction. The hormone concentration of placental tissue was determined following Blottner et al. [40]. The lower detection limit for T, progesterone, E2, and total estrogens was 0.05 ng/mL, 0.5 ng/mL, 20.0 pg/mL and 20.0 pg/mL in plasma, and 0.025 ng/g, 2.5 ng/g, 100 pg/g and 100 pg/g in placenta, respectively. The plasma intra- and interassay coefficient of variations were < 10%.

### Western blot analysis

Equal amounts of placental protein extracts (60 μg protein per lane) were denatured and then separated by a 10% SDS-PAGE. Subsequently, proteins were transferred to a nitrocellulose membrane using semi-dry blotting (Biometra, Göttingen, Germany). For antigen detection, membranes were incubated with primary antibodies goat anti-human aromatase *CYP19A1* [#8799 (1:1,000), Cell Signaling, Frankfurt am Main, Germany], and mouse anti-*GAPDH* [AM4300 (1:10,000), Ambion Inc./Life Technologies, Darmstadt, Germany] over night at 4°C. *GAPDH* protein expression was used as invariant control. For the detection of primary antibodies, membranes were incubated with donkey anti-goat antibody conjugated with IRDye 800CW and donkey anti-mouse antibody conjugated with IRDye 680RD (1:10,000 each; LI-COR Biosciences GmbH, Bad Homburg, Germany) for one hour at room temperature. For signal detection and densitometric analysis, the Odyssey Infrared Detection and Imaging System and the Odyssey application software v3.0 (LI-COR Biotechnology, Bad Homburg, Germany) were used, respectively.

### Assessment of putative hormone-responsive transcription factor binding elements

The UCSC Genome Browser on Human Mar. 2006 (NCBI36/hg18) Assembly of the Encyclopedia of DNA elements (ENCODE) consortium (http://genome.ucsc.edu/) was used for the analysis of putative hormone-responsive elements of the androgen receptor and the estrogen receptors alpha and beta [43]. The validated genes were investigated for HMR (human, mouse, rat) conserved transcription factor binding sites within the nearest neighboring gene upstream to the nearest neighboring gene down-stream of the respective strand.

### Statistical analysis

For statistical analysis, a two-way ANOVA approach was used to assess the factors n-3 LCPUFA intervention [(N3-M + F, n = 21) vs. (Con-M + F, n = 20)], offspring sex [(Con/N3-M, n = 20) vs. (Con/N3-F, n = 21)], and their interactions. With the *post*-*hoc* test Holm-Sidak pairwise comparisons of the intervention and control group stratified for sex were conducted: group 1: placentas of female offspring in the control group (Con-F, n = 11); group 2: placentas of male offspring in the control group (Con-M, n = 9); group 3: placentas from female offspring in the n-3 LCPUFA intervention group (N3-F, n = 10); group 4: and placentas from male offspring in the n-3 LCPUFA intervention group (N3-M, n = 11). Normally distributed variables, as assessed by Shapiro-Wilk test, were analyzed by two-way ANOVA. Sex steroid hormone data and other not normally distributed variables, or variables that violated homoscedascity, were analogously analyzed by a two-way ANOVA on ranks. Significant results of two-way ANOVA and two-way ANOVA on ranks were further analyzed by pairwise comparison with Holm-Sidak *post*-*hoc* test. Two-sided *P*-values < 0.05 were considered as significant. All tests were carried out with Sigma Plot 11.0 (Systat Software GmbH, Erkrath Germany). For bivariate correlation analysis, non-parametric Spearman-rho correlation coefficient (Rs) and two-sided significance were calculated with IBM SPSS Statistics 19 (IBM Deutschland GmbH, Ehningen, Germany). *P*-values < 0.05 were considered as significant correlations, and correlation coefficients between 0.0 - 0.4, 0.4 - 0.7, and 0.7 - 1.0 were considered as weak, moderate, and strong correlations, respectively.

## Results

### Maternal and offspring clinical characteristics of the INFAT subpopulations

To ensure that the main clinical characteristics of INFAT study subpopulation mothers and their offspring, and the compliance of the n-3 LCPUFA intervention are representative for the respective data of the whole INFAT study [6, 23], we analyzed the subpopulations according their usage in the DNA microarray approach and validation experiments stratified for sex (Additional file 1: Table S1 and S2). The analyses of clinical characteristics and LCPUFA biomarker of maternal RBC at week-15 of pregnancy (baseline) showed no significant differences. However, for pregnant mothers at week-32 of pregnancy and offspring cord RBC significantly lower n-6/n-3 LCPUFA ratio in the n-3 LCPUFA intervention compared to the control group were observed, as already reported for the whole INFAT study [6, 23]. These data of the subpopulations in the present study reflect the published excellent compliance of the INFAT study and clearly demonstrate that the n-3 LCPUFA intervention reduces the n-6/n-3 LCPUFA ratio as expected. This confirms that pregnant women with a normal dietary record are not in a state of dietary n-3 LCPUFA saturation, since they respond to the n-3 LCPUFA intervention as shown by increased levels of n-3 LCPUFA in maternal RBC and offspring cord RBC compared to the control group [22, 44].

### Placentas show sexually differential gene expression *per se*

**Figure 1:**
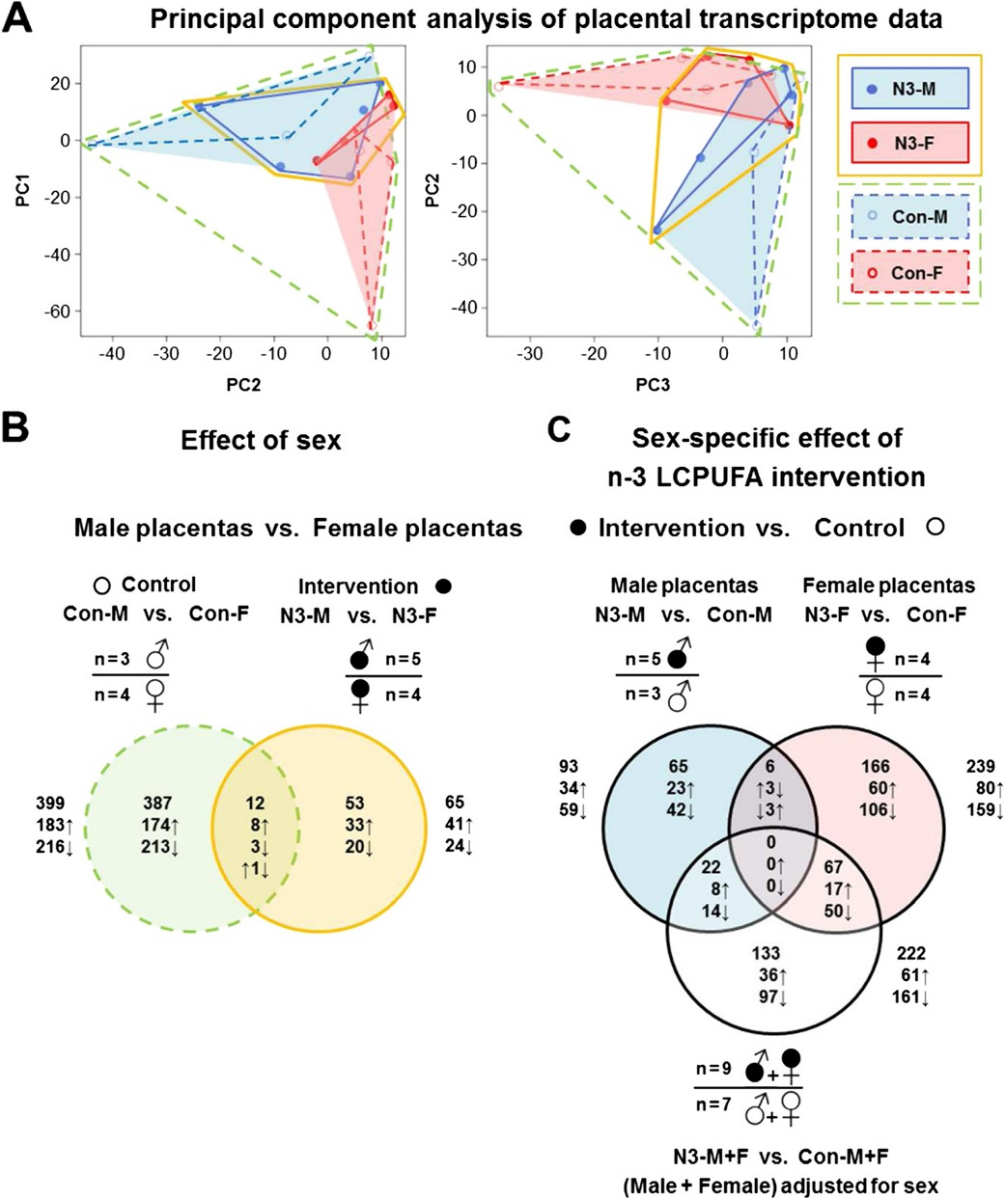
**Principal component analysis (PCA) and Venn diagrams of significantly regulated placental transcripts. (A)** PCA of gene expression data from DNA microarrays. Each circle represents one analyzed placenta (n = 16) projected on the first three principal components (PC): empty circles, control group (Con); filled circles, n-3 LCPUFA intervention group (N3); red circles, female offspring; blue circles, male offspring. Lines connect outer values of the respective placenta groups: green stippled, control placentas; amber, all N3 placentas; red and stippled red, female placentas of N3 and Con, respectively; blue and stippled blue, male placentas of N3 and Con, respectively. Variability of data points for all female and male placentas is represented by red and blue areas, respectively, enclosing corresponding outer values. **(B,C)** Shown Venn diagrams compare the number and intersections of significantly regulated transcripts (+1.5 ≤ FC ≤ −1.5, *P* < 0.05) identified by DNA microarray analysis for the various data sets. **(B)** Green and amber colored Venn diagrams represent the number of significantly regulated transcripts between male and female placentas in Con [(Con-M; n = 3) vs. (Con-F; n = 4)] and N3 [(N3-M; n = 5) vs. (N3-F; n = 5)], respectively. **(C)** Red and blue colored Venn diagrams show the number of significantly regulated transcripts between N3 and Con for male [(N3-M; n = 5) vs. (Con-M; n = 3)] and female placentas [(N3-F; n = 4) vs. (Con-F; n =4)], respectively, and for male and female placentas together and adjusted for offspring sex [(N3-M + F; n = 9) vs. (Con-M + F; n = 7)]. White symbols, Con; black symbols, N3; Con-F, female placentas from Con; Con-M, male placentas from Con; N3-F, female placentas from N3; N3-M, male placentas from N3; arrows up or down depict higher or lower expression related to the respective group.

A DNA microarray approach was applied to 16 placentas from male and female offspring of the INFAT study subpopulation to evaluate sexually differential gene expression *per se* and its responsiveness to maternal dietary n-3 LCPUFA intervention during pregnancy. First, the extent of the effects intervention and sex was assessed in an unsupervised approach for all genes by a principal component analysis (PCA) of the entire normalized array data (Figure 1A). For the data of the first 3 components highlighting intervention and offspring sex as shown in the plots of PC1 vs. PC2 and PC2 vs. PC3 (Figure 1A), the data points neither for intervention nor for offspring sex were clearly aligned with one of these principal components. This result was not surprising, since placental gene expression is a highly complex system with a large number of regulating factors which may also depend on variable maternal parameters. Accordingly, it cannot be expected to find offspring sex to be dominant enough to result in a strong separation in an analysis taking into account all genes present on the DNA microarray. However, visual inspection and highlighting the lines connecting the outer values of the respective data sets, which (i) differentiate between all placentas of the control and intervention group, and (ii) subdivide further these groups by sex, point to a sex-specific effect within the group *per se* and upon the n-3 LCPUFA intervention (Figure 1A).

Next, DNA microarray data were analyzed to identifiy significantly regulated genes considering the effects of n-3 LCPUFA intervention and sex using the following six different comparisons and the MADMAX program. For offspring sex, male placentas were compared to female placentas separately in the (i) control group [(Con-M, n = 3) vs. (Con-F, n = 4)] and (ii) intervention group [(N3-M, n = 5) vs. (N3-F, n = 4)]. To analyze the effect of n-3 LCPUFA intervention, pooled data from male and female placentas of the intervention group were compared to pooled data from male and female placentas of the control group (iii) without adjusting for offspring sex [(N3, n = 9) vs. (Con, n = 7)] and (iv) with adjusting for offspring sex [(N3-M + F, n = 9) vs. (Con-M + F, n = 7)] by using a model including a sex term. Moreover, to identify sex-specific effects of the intervention, the DNA microarray data were stratified for offspring sex. The factor intervention was analyzed (v) in female [(N3-F, n = 4) vs. (Con-F, n = 4)] and (vi) male placentas [(N3-M, n = 5) vs. (Con-M, n = 3)]. Gene expression fold changes (FC) of ≤ −1.5 and ≥ +1.5 (*P* < 0.05) were considered as statistically significant. The main results of these analyses are described as follows and illustrated by Venn diagrams in Figure 1B and C.

We first assessed whether placental gene expression differences exist *per se* between the sexes independent of the n-3 LCPUFA intervention by analyzing the data set (Con-M vs. Con-F) of the control group (Figure 1B). 399 significantly differentially expressed genes were identified. 216 (54.1%) genes showed higher expression in female placentas, whereas 183 (45.9%) genes were higher expressed in male placentas. In the data set (N3-M vs. N3-F) of the intervention group, only 65 differentially expressed genes were found (Figure 1B), and more genes were significantly higher expressed in male [41 (63.1%)] than female placentas [24 (36.9%)]. Furthermore, 12 genes were only in common for both groups as depicted in the Venn diagram intersection area. The remaining 387 (control) and 53 (intervention) genes showed sexually dimorphic expression in a group-restricted manner. For chromosomal localization of the genes in the intersection area, the genes for *DDX3Y*, *EIF1AY*, *KDM5D*, *RPS4Y1*, *USP9Y*, *UTY*, and *ZFY* are located on Y-chromosome, the genes for *HDHD1A*, *KDM6A*, and *ZNF711* are on X-chromosome, and the genes *LIMCH1* and *SSB* are located on autosomes. The genes *DDX3Y*, *EIF1AY*, *KDM5D*, *RPS4Y1*, *USP9Y*, *UTY*, *ZFY*, and *ZNF71* showed significantly higher expression in male than female placentas, whereas the genes *HDHD1A*, *KDM6A*, and *SSB* were significantly higher expressed in female than male placentas in both the control group and the intervention group. For *LIMCH1*, an opposing gene expression between the groups was observed (Additional file 1: Table S4 provides expression fold changes of some of these genes and their relation to placental transcriptome studies by Sood et al. [15] and Tsai et al [37]). Interestingly, the X-chromosome-linked genes *HDHD1A* and *KDM6A* were nearly two-fold higher expressed in female than male placentas confirming reports that they escape X-inactivation in humans [45–47].

### Importance of offspring sex in placental gene expression analyses

The reason to include the term sex in our statistical analysis was based on our initially interesting observation that in the data set (N3 vs. Con; not adjusted for offspring sex) only 22 genes in total were significantly regulated between the intervention and control group (10 up-regulated genes, 12 down-regulated genes). However, after adjusting this data set for offspring sex (N3-M + F vs. Con-M + F), 222 significantly regulated genes upon intervention were found (Figure 1C). Importantly, more genes were down-regulated [161 (72.5%)] than up-regulated [61 (27.5%)] upon intervention.

The evaluation whether the intervention impacts gene expression differently in male and female placentas (N3-F vs. Con-F) led to the identification of 239 n-3 LCPUFA-regulated genes in female placentas (N3-F vs. Con-F) [80 (33.5%) up-regulated genes; 159 (66.5%) down-regulated genes], whereas for male placentas (N3-M vs. Con-M) only 93 genes were found [34 (36.6%) up-regulated genes; 59 (63.4%) down-regulated genes]. Most of the genes were unique for one of the three data sets: 65 genes (N3-M vs. Con-M), 133 genes (N3-M + F vs. Con-M + F), and 166 genes (N3-F vs. Con-F) as shown in Figure 1C.

For n-3 LCPUFA-regulated genes common in two data sets, only 22 genes (N3-M + F vs. Con-M + F; N3-M vs. Con-M), and 67 genes (N3-M + F vs. Con-M + F; N3-F vs. Con-F) without changes of regulation direction for both respective data sets were found. In contrast, for the data sets (N3-M vs. Con-M) and (N3-F vs. Con-F), the only common genes with significantly differential expression were *CKS2*, *C8orf59*, *EPS8L2*, *GALNT11*, *LIMCH1*, and *STRA6*, but these genes showed an inverse regulation. n-3 LCPUFA-regulated genes common to all three analyzed data sets were not found.

### Identification of n-3 LCPUFA-responsive genes and pathways

Since there was a lack of data in the literature on placental transcriptome analysis with regard to n-3 LCPUFA intervention during pregnancy, the expression of known classical LCPUFA-regulated genes that were already predominantly described in non-placental tissues, such as adipose tissue and liver [14, 48, 49], were evaluated by our placental transcriptome data. Surprisingly, only a few of those genes showed minor placental expression changes in response to n-3 LCPUFA intervention (for details see Additional file 1: Text S1, Tables S5-S7). We therefore searched for alternative n-3 LCPUFA-responsive genes and biological pathways by applying *Pathvisio* pathway analysis to our transcriptome data sets. Table 1 shows the top ten ranked pathways as follows, which (i) had a significantly overrepresented number of regulated genes (Z-score > 0) according to *Pathvisio*, (ii) were present in at least two of the five analyzed DNA microarray data sets, and (iii) included more than four significantly regulated genes per pathway: ‘Cell cycle A’, ‘Insulin signaling’, ‘Oocyte meiosis’, ‘Cell cycle B’, ‘Cytokine-cytokine receptor interaction’, ‘Adipogenesis’, ‘B cell receptor signaling’, ‘Wnt signaling’, ‘*TGFB* receptor signaling’, and ‘Myometrial relaxation and contraction’. In addition, 11 different genes showing each an overlap for significant expression regulation with regard to both the sex and the n-3 LCPUFA intervention in at least one specific pathway were identified (Table 1).

**Table 1:** **Summary of pathways containing overrepresented differentially expressed genes from DNA microarray data sets analyzed by offspring sex and n-3 LCPUFA intervention**

Pathways M/TP	Sex		n-3 LCPUFA intervention			Genes overlapping for sex & n-3 LCPUFA
	Con-M vs. Con-F	N3-M vs. N3-F	N3-F vs. Con-F	N3-M vs. Con-M	N3-M + F vs. Con-M + F ^§^	
**Cell cycle A**	Z: 1.2; *GSK3B*, *CDKN1A*, *YWHAB*, *CCNH*, ***CDK6***	-	Z: 3.1; ***TGFB1***, *SMC3*, ***MAD2L1***, ***ANAPC4***, ***CDK6***, ***CDK1***	-	Z: 3.0; *CDK4*, ***ANAPC4***, *CCNB1*, *MCM3*, *DBF4*	***CDK6***
112/131						
Insulin signaling	Z: 2.9; *SH2B2*, *TRIP10*, *GSK3B*, *FASN*, *CALM1*, *PRKX*	-	Z: 2.1; *PRKACB, SH2B2, RHOQ, PPP1R3D, PYGL*	Z: 4.6; *TRIP10*, *HK2*, *CALM1*, *IRS2*, *SH2B2*	-	*SH2B2*, *TRIP10*, *CALM1*
126/147						
Oocyte meiosis^†^	Z: 2.4; *PRKX*, *PPP3CA*, *CALM1*, *CAMK2G*, *YWHAB*	-	Z: 3.3; *PRKACB*, ***CDK1***, ***ANAPC4***, *SMC3*, ***MAD2L1***, *PPP3CA*	-	Z: 2.4; *PPP2R1A,* ***ANAPC4***, *CCNB1, FBXO5*	*PPP3CA*
103/122						
**Cell cycle B**	Z: 1.1; *GSK3B*, ***CDK6***, *CDKN1A*, *CCNH*	-	Z:3.2; ***TGFB1***, ***CDK6***, ***CDK1***, ***MAD2L1***, ***HDAC5***	-	Z: 3.0; *CDK4*, *CCNB1*, *DBF4*, *MCM3*	***CDK6***
81/94						
Cytokine-cytokine receptor interaction^†^	-	Z: 2.6; *CXCR7*, *CCR5*, *LEP*, *BMP2*	Z: 0.7; *IL8*, *FLT4*, ***TGFB1***, *CSF3R CCL13*,	Z: 2.2; *CXCR7*, *TNFRSF21*, *PRL*, *NGFR*	-	*CXCR7*
225/267						
Adipogenesis^†^	Z: 4.5; *LIFR*, *IL6ST*, ***DVL1***, *FRZB*, *ID3*, *SREBF1*, *MBNL1*, *NRIP1*, *CDKN1A*, ***LPL***, *LPIN1*	-	Z: 2.2; ***LPL***, ***TGFB1***, *MEF2C*, *BMP1*, *FRZB*	-	-	***LPL***, *FRZB*
117/131						
B cell receptor signaling^†^	Z: 1.5; *RAP2A*, *PIK3AP1*, *SH2B2*, *ATP2B4*, *GSK3B*, *ACTR2*, *RASGRP3*, ***CDK6***, *PPP3CA*	-	Z:2.4; *PIK3AP1*, *SH2B2*, *PP3CA*, ***CDK6*** **,** ***HDAC5***, *BCL6*	-	-	*PIK3AP1*, *SH2B2*, *PP3CA*, ***CDK6***
144/159						
**Wnt signaling**	Z: 2.5; ***SFRP1***, *GSK3B*, *PRKX*, *TBL1X*, *ROCK1*, *CAMK2G*, *PPP3CA*, ***DVL1***, *FZD6*	-	Z: 1.8; ***SFRP1***, ***LRP6***, *PRKACB*, *TBL1X*, *PPP3CA*	-	-	***SFRP1***, *TBL1X*, *PPP3CA*
139/162						
***TGFB*** **receptor signaling** ^**†**^	Z: 1.1; *ROCK1*, ***DVL1***, *CAMK2G*, *CDKN1A*, ***CDK6***, *YAP1*	-	Z: 2.6; ***TGFB1***, ***CDK1***, ***ANAPC4***, ***CDK6***, *MEF2C*, *ZEB1*	-	-	***CDK6***
134/152						
Myometrial relaxation & contraction	Z: 2.4; *GNB1*, *GUCY1A3*, *ACTB*, *CALM1*, *RGS5*, *YWHAB*, *CAMK2G*	-	-	Z: 4.1; *IGFBP1*, *PRKAR1B*, *CXCR7*, *RXFP1*, *GNG8*	-	
146/161						

M, number of genes measured by DNA microarray and annotated in the pathway; TP, total number of genes annotated in the pathway; ^§^adjusted for offspring sex; Z, Z-score = (significantly regulated genes – expected genes)/(standard deviation of significantly regulated genes); −, pathways without positive Z-scores, or if less than four genes are significantly regulated in the pathway; ^†^pathway includes significantly overrepresented differentially expressed genes that are also present in the pathways ‘Cell cycle A’ and ‘Cell cycle B’. Pathways and genes selected for biological validation are marked in bold. Con-M, placentas of male offspring in the control group; Con-F, placentas of female offspring in the control group; N3-M, placentas of male offspring in the n-3 LCPUFA intervention group; N3-F, placentas of female offspring in the n-3 LCPUFA intervention group.

**Figure 2:**
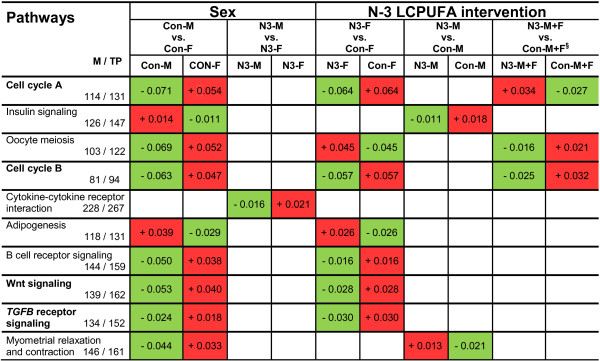
**Difference of mean centroids between the analysis groups indicating the direction of gene expression changes in the pathways.** Mean centroid values of the analyzed groups are shown for the different data sets. Positive and negative mean centroid values represent up-regulation (red) and down-regulation (green) of gene expression, respectively. M, number of genes measured by DNA microarray and annotated in the pathway; TP, total number of genes annotated in the pathway; §, adjusted for offspring sex; Con-M, placentas of male offspring in the control group; Con-F, placentas of female offspring in the control group; N3-M, placentas of male offspring in the n-3 LCPUFA intervention group; N3-F, placentas of female offspring in the n-3 LCPUFA intervention group.

To characterize the general direction of gene expression changes in the pathways with regard to effects of sex and intervention, we performed mean centroid value analyses for the specific pathways (Figure 2). Since positive and negative mean centroid values suggest higher and lower gene expression in all analyzed genes in the pathway, respectively, the following main findings for sex and intervention effects, considering mean centroid values > + 0.01 and < − 0.01, were obtained. The data set (Con-M vs. Con-F) indicated for the identified pathways ‘Cell cycle A’, ‘Oocyte meiosis’, ‘Cell cycle B’, ‘B cell receptor signaling’, ‘Wnt signaling’, ‘*TGFB* receptor signaling’, and ‘Myometrial relaxation and contraction’ a down-regulation in male compared to female placentas, whereas for the pathways ‘Adipogenesis’ and ‘Insulin signaling’ the opposite direction was observed. For the effect of intervention on female placentas specifically (N-3-F vs. Con-F), gene expression in the pathways ‘Cell cycle A’, ‘Cell cycle B’, ‘B cell receptor signaling’, ‘Wnt signaling’, and ‘*TGFB* receptor signaling’ appeared to be down-regulated compared to controls, whereas for the pathways ‘Oocyte meiosis’ and ‘Adipogenesis’ an up-regulated gene expression was observed. For male placentas (N-3-M vs. Con-M), the analysis pointed to a down-regulation of gene expression in the pathway ‘Insulin signaling’ and an up-regulated gene expression in the pathway ‘Myometrial relaxation and contraction’.

### RT-qPCR validation of placental genes

**Figure 3:**
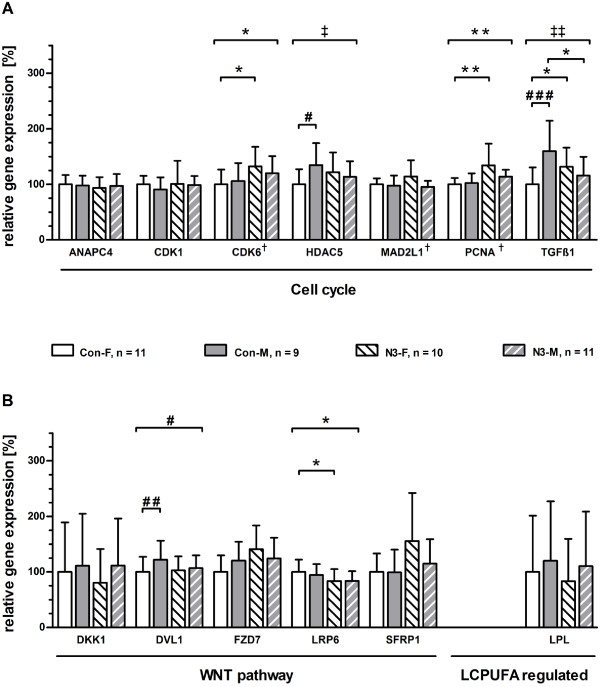
**Biological validation of placental genes in ‘Cell cycle’, ‘Wnt signaling’ and ‘LCPUFA regulation’.** Expression levels of genes representing the pathways ‘Cell cycle’ and ‘Wnt signaling’, as well as ‘LCPUFA regulation’ are shown. The data bars for the four analyzed groups (Con-F, Con-M, N3-F, N3-M) are differently shaded as depicted by the respective boxes between **panel A and B**. The expression level of female placentas of the control group (Con-F) was assigned an arbitrary value of 100% and all other analyzed groups (Con-M, N3-F and N3-M) were depicted relative to Con-F. Data are presented as mean relative gene expression in % + 95% confidence interval. Statistically significant effects of specific factors were marked as follows. #, offspring sex; *, n-3 LCPUFA treatment; ^‡^, interactions. #, * or ^‡^ *P* < 0.05; # #, * * or ^‡‡^, *P* < 0.01; # # # or * * *, *P* < 0.001. ^†^, two-way ANOVA on ranks. Con-F, female placentas from the control group; Con-M, male placentas from the control group; N3-F, female placentas from the n-3 LCPUFA intervention group; N3-M, male placentas from the n-3 LCPUFA intervention group. n, number of validated placentas per group: Con-F, n = 11; Con-M, n = 9; N3-F, n =10; N3-M, n = 11.

For a gross validation by RT-qPCR of our findings on n-3 LCPUFA-responsive genes and biological pathways, RNAs from placentas of the control (Con-F, n = 10; Con-M, n = 11) and intervention (N3-F, n = 11; N3-M, n = 9) group were analyzed individually. We focused on the genes for *ANAPC4*, *CDK1*, *CDK6*, *HDAC5*, *MAD2L1*, and *TGFB1*, which were found to be significantly expressed in the two biological pathways ‘Cell cycle A’ and ‘Cell cycle B’, but additionally also in other presented pathways of this study (Table 1). Furthermore, the gene for *PCNA*, known as general cell proliferation marker [50], was also validated, since it almost reached the significant FC level in our transcriptome analysis (N3-F vs. Con-F; FC: +1.4; *P* = 0.004). As shown in Figure 3, apart from the n-3 LCPUFA-responsive genes, we validated the following genes present in the respective pathways [51, 52]: *LPL* and *TGFB1* (upstream regulators of cell cycle), *ANAPC4* (‘*TGFB* receptor signaling’), and *DKK1*, *DVL1*, *FZD7*, *LRP6*, and *SFRP1* (‘Wnt signaling’). For the ‘Cell cycle’ (Figure 3A), CDK6 and *PCNA* gene expression were significantly higher in the intervention than control group by applying two-way ANOVA on ranks. Applying *post*-*hoc* tests showed only significant differences (expressed as mean ± SD in % of Con-F) between the female placentas of the intervention and control group [N3-F vs. Con-F: *CDK6* 132 ± 49% vs. 100 ± 37%, *P*_N3-F vs. Con-F_ = 0.046; *PCNA* 134 ± 55% vs. 100 ± 17%, *P*_N3-F vs. Con-F_ = 0.005]. The two-way ANOVAs for *HDAC5* and *TGFB1* revealed significant interactions between the intervention and offspring sex. The *post*-*hoc* test for *HDAC5* demonstrated significantly higher mRNA expression in male than female placentas of the control group [Con-M vs. Con-F: 135 ± 52% vs. 100 ± 41%, *P*_Con-M vs. Con-F_ = 0.016], but not in the intervention group. Furthermore, *post*-*hoc* analysis for *TGFB1* showed also a significant difference between male and female placentas in the control group (Con-M vs. Con-F: 160 ± 71% vs. 100 ± 46%, *P*_Con-M vs. Con-F_ < 0.001), but not in the intervention group. At the same time, *TGFB1* expression in female placentas was significantly higher by 32% in the intervention than control group (N3-F vs. Con-F: 132 ± 48% vs. 100 ± 46%, *P*_N3-F vs. Con-F_ = 0.018), whereas *TGFB1* expression was significantly lower by 44% in male placentas of the intervention group compared to the control group (N3-M vs. Con-M: 116 ± 50% vs. 160 ± 71%, *P*_N3-M vs. Con-M_ = 0.016). No significant difference in expression was observed for *ANAPC4*, *CDK1*, and *MAD2L1*.

For the ‘Wnt signaling’ genes *DVL1* and *LRP6* (Figure 3B), two-way ANOVA analyses revealed significant expression differences between the intervention and control group, as well as significant differences between male and female placentas, respectively [*DVL1* (Con-M vs. Con-F): 122 ± 41% vs. 100 ± 41%, *P*_Con-M vs. Con-F_ = 0.009; *LRP6* (N3-F vs. Con-F): 84 ± 30% vs. 100 ± 33%, *P*_N3-F vs. Con-F_ = 0.049]. There was no significant gene expression difference measured for *DKK1*, *FZD7*, *LPL*, and *SFRP1*.

### Sex steroid hormone levels in placental tissue and cord plasma

**Figure 4:**
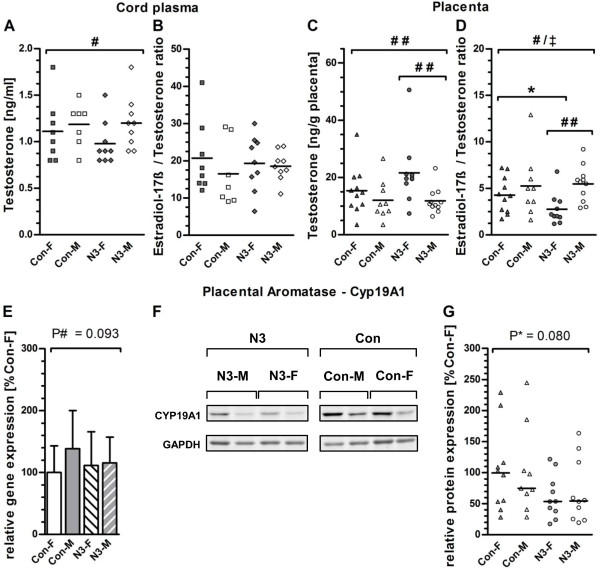
**Sex steroid levels in placenta and cord plasma, and aromatase gene and protein expression.** Testosterone (T) levels and estradiol-17-beta/testosterone (E2/T) ratio in cord plasma **(A,B)** and placenta **(C,D)** are presented as scatter plots with median. **(E)** RT-qPCR data for *CYP19A1*(aromatase) gene expression for the female placentas of the control group (Con-F) are assigned to an arbitrary value of 100% and values for all other analyzed groups (Con-M, N3-F, and N3-M) were depicted relative to Con-F. Data are presented as mean relative gene expression in % + the 95% confidence interval. **(F)** Representive data from Western blot analyses for *CYP19A1* (51 kDa) and *GAPDH* (36 kDa) are shown. *GAPDH* was used for normalization. **(G)** *CYP19A1* protein expression data are summarized in a scatter blot with median. Con-F samples are assigned to an arbitrary value of 100% and all other analyzed groups were depicted relative to Con-F. Statistical significance was calculated by parametric two-way ANOVA for RT-qPCR data and non-parametric two-way ANOVA on ranks for all other data. Holm-Sidak *post-hoc* test was used for group comparisons. Statistically significant effects of specific factors were marked as follows. #, offspring sex; *, n-3 LCPUFA treatment; ^‡^, interactions. #, * or ^‡^, *P* < 0.05; # # or * * *P* < 0.01. Trends (*P* < 0.1 – 0.05) are shown as the actual *P* value. Con-F and Con-M, female and male offspring in the control group, respectively; N3-F and N3-M, female and male offspring in the n-3 LCPUFA intervention group, respectively. Number of analyzed placentas per group: Con-F, n = 11; Con-M, n = 9; N3-F, n = 10, and N3-M, n = 11. Number of analyzed cord plasma samples per group: Con-F, n = 8; Con-M, n = 7; N3-F, n = 9; N3-M, n = 9.

Since sex steroid hormones are major determinants of sexual dimorphism [17, 53], sex steroid hormone levels were explored whether they contributed to the sex-specific placental gene expression in our study. Free and conjugated E2, free and conjugated total estrogen, T, progesterone, and the E2/T ratio in placental tissue (n = 41) and cord plasma (n = 34) were measured. For T, a significant effect of offspring sex in cord plasma and placenta was detected by two-way ANOVA analysis on ranks (Figure 4A and C), whereas no significant effects were observed for the other analyzed sex steroid hormones progesterone and estrogens in cord plasma or placenta considering intervention or offspring sex (see Additional file 1: Table S8). *Post*-*hoc* analysis for cord plasma T (Figure 4A) detected a significantly lower T level by 12.5% in female than male placentas independent of intervention [Con-M: 1.3 (1.0 - 1.3) ng/ml; Con-F: 1.1 (0.8 - 1.3) ng/ml; N3-M: 1.2 (1.0 - 1.4) ng/ml; N3-F: 0.9 (0.8 - 1.1) ng/ml; (Con/N3-M) vs. (Con/N3-F); *P*# = 0.049]. However, *post*-*hoc* analysis for placental T revealed significantly higher levels by 54.3% in female compared to male placentas from the intervention group [median (IQR): 19.7 (16.6 - 22.6) ng/g N3-F placenta vs. 10.7 (9.7 - 14.4) ng/g N3-M placenta; *P*_N3-M vs. N3-F_ = 0.008], but no significant differences in control placentas as shown in Figure 4C. To further analyze the differences in hormone levels between the groups, the E2/T ratio was calculated and used as activity index for aromatase (*CYP19A1*) which executes the transformation of T to E2 [54]. Interestingly, significant effects of offspring sex and interaction of sex with the intervention were observed for the placental E2/T ratio using two-way ANOVA on the ranks (Figure 4D). *Post*-*hoc* analysis showed a significantly higher E2/T ratio by 171.4% in male than female placentas upon n-3 LCPUFA treatment [5.7 (3.6 - 6.3) ng/g N3-M vs. 2.1 (1.7 - 3.7) ng/g N3; *P*_N3-M vs. N3-F_ = 0.002], but not in the control. Additionally, *post*-*hoc* analysis revealed a significantly lower E2/T ratio by 49.9% for female placentas of the intervention than control group [2.1 (1.7-3.7) ng/g N3-F vs. 4.2 (2.5-6.1) ng/g Con-F; *P*_N3-F vs. Con-F_ = 0.042] reflecting significantly higher T levels in N3-F compared to Con-F placentas without significant difference for E2. For male placentas (N3-M vs. Con-M), however, no significantly different E2/T ratio was found. Moreover, cord plasma E2/T ratios between the analyzed groups were also not significantly different (Figure 4B).

### Placental gene and protein expression for *CYP19A1*

Considering that T levels in female placentas of the intervention group were significantly increased, we hypothesized that increased T levels might be due reduced expression of aromatase *CYP19A1* that slows down the conversion of T to E2 [54]. Although contradictory data were reported from humans and animals studies on the impact of n-3 LCPUFA on circulating T levels [55–59], it was hypothesized that n-3 LCPUFAs influence T production in adult pig testis via their impact on PGE_2_ synthesis or their regulation of PPAR-mediated stearidogenesis [55]. Thus, since n-3 LCPUFA intervention reduces n-6-LCPUFA and their metabolites, such as PGE_2_ [6, 23, 44], changes in T levels may be mediated by altered *CYP19A1* levels. Therefore, placental mRNA and protein expression for *CYP19A1* were determined by RT-qPCR and Western blot analyses, respectively. For *CYP19A1* mRNA levels, no significant changes related to effects of sex or n-3 LCPUFA intervention were observed, however, a trend (*P*^#^ = 0.093) for higher mRNA levels in male placentas was observed (Figure 4E). For aromatase protein, a trend for lower levels in the intervention than in the control group was found, but this difference was also not statistically significant (Figure 4F and G).

### Correlation of sex steroid hormone levels in placenta and cord plasma with regulated placental expression of specific genes

Placental testosterone levels did not significantly correlate with cord plasma T levels (Rs = − 0.031, *P* = 0.865, n = 33). However, the E2/T ratio showed a positive, moderate correlation between placental tissue and cord plasma (Rs = 0.424, *P* = 0.014, n = 33). There were no significant correlations for the genes *CDK6*, *FZD7*, *HDAC5*, *PCNA*, and *TGFB1* (Table 2). Only mRNA levels for the ‘Wnt signaling’ genes *DVL1* and *LRP6* showed significantly positive correlations with placental E2/T ratio in a weak and moderate manner, respectively.

**Table 2:** **Correlation analyses of placental and cord plasma testosterone levels, and placental estradiol-17ß with validated sex-specific mRNA expression differences of specific genes**

	***CDK6***			***PCNA***			***HDAC5***			***LRP6***		
	PL_T	PL_E2/T	CP_T	PL_T	PL_E2/T	CP_T	PL_T	PL_E2/T	CP_T	PL_T	PL_E2/T	CP_T
**Rs**	−0.06	0.19	0.08	−0.13	−0.01	−0.03	−0.09	0.18	−0.17	−0.28	**0.48**	0.13
***P***	0.734	0.254	0.658	0.422	0.969	0.864	0.591	0.268	0.339	0.080	**0.002**	0.476
**N**	38	38	31	41	41	33	41	41	33	41	**41**	33
	***DVL1***			***FZD7***			***TGFB1***					
	**PL_T**	**PL_E2/T**	**CP_T**	**PL_T**	**PL_E2/T**	**CP_T**	**PL_T**	**PL_E2/T**	**CP_T**			
**Rs**	−0.25	**0.32**	0.01	0.07	0.06	−0.21	−0.14	0.11	−0.15			
***P***	0.115	**0.044**	0.955	0.688	0.728	0.233	0.395	0.490	0.422			
**N**	41	**41**	33	41	41	33	41	41	33			

CP_T, cord plasma testosterone; N, number of analyzed individual samples or subjects; *P*, P-value for the correlation; PL_E2/T, placental estradiol-17ß / testosterone ratio; PL_T, placental testosterone; Rs, Spearman-rho correlation coefficient. Bold data represent significant correlations (*P* < 0.05).

To explore further the potential that sex steroid hormones are implicated directly in the observed sex-specific gene regulation via their hormone receptors, we assessed if the analyzed target genes depict putative hormone-responsive elements for the estrogen and androgen receptors between the nearest neighboring gene upstream to the nearest neighboring gene down-stream of the respective strand. In our search, only estrogen receptor alpha response elements were found, but no androgen receptor elements. For the genes involved in cell cycle (*ANAPC4*, *CDK1*, *CDK6*, *HDAC5*, *MAD2L1*, *PCNA*, and *TGFB1*), only for the genes *CDK6* and *MAD2L1* putative estrogen receptor alpha binding sites were found, whereas for the other genes no respective responsive elements were detected. The binding site for *CDK6* was located in the gene body, whereas the binding site for *MAD2L1* was located about 200 kBb upstream of the gene body.

Interestingly, with regard to the analyzed genes in the pathway ‘Wnt signaling’, for nearly all genes, such as *DVL1*, *FZD7*, *LRP6*, and *SFRP1*, at least one estrogen receptor alpha response element was annotated within their gene body. The same was observed for *LPL*. In addition, for *CYP19A1*, three estrogen receptor alpha response elements were annotated, which were located about 100 and 30 kBp upstream, and about 70 kBp downstream of the gene body.

### Correlation of regulated placental gene expression with offspring weight and body composition

Correlation analyses were conducted to evaluate whether placental differential mRNA expression of specific genes upon intervention might be associated with placental factors, body weight and length, or body composition measurements of offspring up to one year. All parameters with significant correlations are presented in Table 3 and a complete list of all calculations is shown in Additional file 1: Table S9. *CDK6* and *PCNA* mRNA levels showed significantly positive correlations with birth weight and birth weight percentiles. Additionally, *PCNA* was also significantly positively correlated with birth weight/birth length ratio in a weak manner. Both, *DVL1* and *HDAC5* were significantly positively correlated with offspring body weight at one year in a moderate manner (Table 3). No significant correlations were found for placental weight and body composition (see Additional file 1: Table S9).

**Table 3:** **Correlation analyses of significantly regulated mRNA levels of genes with offspring anthropometric measurements at birth and one year of life, and with placental weight**

	***LRP6***	***DVL1***	***PCNA***	***CDK6***	***HDAC5***	***TGFB1***
	**Birth weight**					
**Rs**	0.06	−0.02	**0.31**	**0.33**	0.06	−0.15
***P***	0.719	0.918	**0.049**	**0.042**	0.717	0.339
**N**	41	41	**41**	**38**	41	41
	**Birth weight percentiles**					
**Rs**	0.17	−0.04	**0.49**	**0.36**	0.04	−0.08
***P***	0.295	0.795	**0.002**	**0.023**	0.826	0.607
**N**	41	41	**38**	**41**	41	41
	**Birth weight-to-length ratio**					
**Rs**	0.06	0.01	**0.32**	0.26	0.04	−0.19
***P***	0.721	0.931	**0.039**	0.111	0.825	0.232
**N**	41	41	**41**	38	41	41
	**Birth weight-to-placental weight ratio**					
**Rs**	−0.12	0.16	0.08	0.26	0.28	0.18
***P***	0.471	0.325	0.626	0.111	0.079	0.250
**N**	41	41	41	38	41	41
	**Placental weight**					
**Rs**	0.13	−0.15	0.12	−0.09	−0.23	−0.30
***P***	0.402	0.366	0.462	0.595	0.151	0.055
**N**	41	41	41	38	41	41
	**Body weight at year-1**					
**Rs**	0.03	**0.49**	0.15	0.22	**0.48**	0.20
***P***	0.834	**0.001**	0.350	0.193	**0.002**	0.210
**N**	40	**40**	40	37	**40**	40

N, number of analyzed individual samples or subjects; *P*, P-value for the correlation; Rs, Spearman-rho correlation coefficient. Bold data represent significant correlations (*P* < 0.05).

## Discussion

The placenta is indispensable for nutrition and development of the mammalian fetus and therefore recognized as a major target for fetal programming [7, 8]. Males and females show sex-specific differences in organ physiology and prevalence of major diseases, including autoimmune diseases, diabetes, and asthma [18, 19]. The actions of sex steroid hormones have been implicated in the susceptibility to these differences between sexes [10, 17–19]. However, investigations of offspring sex-specific gene expression and responses in the placenta have been widely neglected [9].

The major finding of our study was that placental gene expression is different between male and female offspring of the control group, and that maternal dietary n-3 LCPUFA intervention during pregnancy impacts sex-specifically placental gene expression predominantly in female placentas. Our transcriptome data sets suggest that maternal n-3 LCPUFA intervention counterbalanced mostly the sexually dimorphic gene expression pattern observed in the control group, but also induced the regulation of other genes resulting in sexually differential expression *de novo*. n-3 LCPUFA-responsive genes were enriched in several pathways, such as for ‘Adipogenesis’, ‘Cell cycle’, and ‘Wnt signaling’, consistent with the reported importance of the cell cycle and Wnt signaling for placental development and physiology [60–62]. In addition, the mean centroid value analyses of pathway-specific gene expression and RT-qPCR data also suggest a counter-regulation of selected genes and pathways upon n-3 LCPUFA. Overall, this supports our transcriptome data on significantly differential gene expression, where upon n-3 LCPUFA intervention in general more genes were found to be down-regulated than up-regulated in female placentas. In the context of adipose tissue growth, it is important to note that the the general direction of gene expression for the pathway ‘Adipogenesis’ was indicated to be up-regulated in female placentas from the intervention group. The evaluation of offspring sex steroid hormone levels at birth indicated an impact on placental T and E2/T ratio upon intervention, however without strong associations to placental sex-dependent gene expression changes or to the presence of putative hormone-responsive elements in the vicinity of the respective genes. Regarding offspring adiposity risk, significantly moderate associations of placental mRNA levels for *CDK6*, *DVL1*, *HDAC5*, and *PCNA* with offspring birth weight and birth weight percentiles were observed, but not with body composition in the first year of age.

### Sexually dimorphic gene expression in human control placentas

In the present study, a considerable number of identified genes with sexually dimorphic expression are located on autosomes, but as expected, some genes are also located on sex chromosomes. This is in line with Sood et al. [15] describing sex-specifically expressed genes in normal human term placentas. In contrast, in the study of Tsai et al. [37] nearly all 36 identified genes with sex-specific expression in human placenta are located on sex chromosomes (Additional file 1: Table S4 provides genes identified in the present study common with genes in the data sets of Sood et al. [15] and Tsai et al. [37]). Apart from our findings on genes located on autosomes, the expected confirmation of sex-specific expression of genes from sex-chromosomes, serving as an internal control, supports the quality and validity of our DNA microarray experiments [63]. Moreover, our data are consistent with comprehensive gene expression analyses between murine male and female organs that demonstrate sexual dimorphism for genes with rather small fold gene expression differences (1.2 to 2.0), high tissue-specificity, and gene locations on sex chromosomes and autosomes [19].

### Gene expression in female placentas is more responsive to maternal n-3 LCPUFA supplementation than in male placentas

Our finding that it is important to include offspring sex as factor in the analyses led to the identification of a substantially increased number of n-3 LCPUFA-responsive genes. Surprisingly, only a few of these genes showed common regulation in both sexes. Since neither sex-dependent nor sex-independent analyses of the placental transcriptome with regard to n-3 LCPUFA intervention during pregnancy have been published which could be used for the evaluation of our results, we compared our data to transcriptome data on non-placental tissues or cells [64, 65], which also show considerable number of regulated genes between n-3 PUFA intervention and control group in healthy humans. Rudkowska et al. [65] detected among many n-3 PUFA-responsive genes in peripheral blood mononuclear cells only 9 genes that were expressed in common in both adult males and females. Furthermore, Gabory et al. [66] showed that in placentas from offspring mice of mothers, fed control and high-fat diet during pregnancy, only a small portion of genes (11 out of 178) displayed sexually dimorphic gene expression which was common for both dietary groups. These data support our findings despite the tissue and species differences, and the lower numbers of microarrays used per group in our study compared to the studies of Rudkowska et al. [65] and Gabory et al. [66]. In addition, this might also explain why we observed only a few n-3 LCPUFA-responsive genes when placental sex was not considered in the analysis.

Our finding that more genes were significantly regulated in female placenta upon nutritional intervention is in line with other reports on placenta. For example, more genes with significantly regulated placental expression were found in female offspring in response to maternal asthma in humans [67] and maternal diet in mice [68]. Clifton et al. [9] hypothesized that ‘the minimal placental gene alterations of the male placenta may be a mechanism which allows the male fetus to continue growing in an adverse environment’. However, a maternal supplementation of n-3 LCPUFAs during pregnancy was suggested to have beneficial rather than adverse effects [6, 69–71]. Therefore, we propose that the hypothesis of Clifton [9] should be more generalized towards ‘the minimal gene alterations of the male placenta may be a mechanism that allows the male fetus to maintain growth relatively independent of environmental stimuli’. Conversely, the considerable changes in gene expression of female placentas might allow the female fetus to adapt more comprehensively to environmental stimuli. However, the physiological relevance of the extensive adaptation of female placental gene expression to their environment remains to be explored.

### Placental cell cycle-associated *CDK6*and *PCNA*gene expression is correlated with offspring birth weight and birth weight percentiles

In the present study, the biological validation of n-3 LCPUFA-responsive genes, enriched in at least the pathways ‘Cell cycle’ and ‘Wnt signaling’, confirmed the observed sex-specific effects of the n-3 LCPUFA intervention. Although there exist only a few reports on human placental gene expression with regard to maternal n-3 LCPUFA intervention during pregnancy [72, 73], our finding that n-3 LCPUFA may affect biological processes associated with cell cycle and its regulation in the placenta is consistent with these reports. Klingler et al. [72] showed that a nutritional DHA and 5-methyltetrahy-drofolate supplementation during pregnancy increased *PCNA* protein expression exclusively in trophoblast cells of the human term placenta. For a human trophoblast cell line, Johansen et al. [73] showed that DHA, but not EPA, stimulated cell proliferation. Cell proliferation has utmost importance for development and maintenance of the human placenta as shown for the cytotrophoblast and syncytiotrophoblast [60, 61], and it has to be tightly controlled by numerous factors [61] including *CDK6* and *PCNA* [50, 74]. Although for human placenta increased proliferation is rather associated with maternal preeclampsia, anemia, or diabetes [60, 75, 76], these associations do not apply to our study, where the mothers who donated the analyzed placentas were healthy without complications during pregnancy [6]. Hypothesizing that changes in the placenta upon n-3 LCPUFA intervention during pregnancy are involved in the programming of offspring obesity risk, we discovered that placental *CDK6* and *PCNA* mRNA levels were positively correlated with offspring birth weight and birth weight percentiles. Interestingly, Zadrozna et al. [77] showed that in intrauterine growth retardation with associated lower birth weight less placental cells of terminal villi expressed *PCNA* accompanied with reduced cytotrophoblast cell proliferation. In contrast, we found that higher *PCNA* gene expression in placental cells correlated with higher birth weight and birth weight percentiles. Although, *CDK6* and *PCNA* gene expression were significantly higher in female placentas of the intervention group, there were no substantial differences found for offspring birth weight and body composition in the first year of age between these groups neither in the INFAT subpopulation nor in the whole INFAT population after adjustment for sex and gestational duration [6]. Our data raise the questions whether the changes in expression of placental genes and pathways reflect (i) a counteractive response to the proposed growth reducing impact of n-3 LCPUFA supplementation on adipose tissue, or (ii) missing strong associations with the analyzed offspring anthropometric data, since n-3 LCPUFA *per se* does not lead to adipose tissue reduction in early human infancy as proposed by the data of Ailhaud and co-workers [4, 5].

### Placenta and cord blood T levels and E2/T ratio show only a minor contribution to placental sexually dimorphic gene expression and sex-specific response to the n-3 LCPUFA intervention

Data on the underlying mechanism(s) mediating sexual dimorphism are scarce in humans and largely based on observations from mouse or rodent models [17, 53]. Sex steroid hormones are thought to provide the initiation of sexual differentiation in somatic tissue, but also genes from the sex chromosomes contribute to differential gene expression between male and female somatic tissues [17]. As a start to get molecular insights into the molecular mechanisms mediating the observed sexually dimorphic gene expression and potential fetal programming effects, in this study, we analyzed sex steroid hormone levels and related significant changes to offspring placental gene expression and body composition.

For placental estrogene and testosterone, our measured T and E2 levels were in the same range as reported by other antibody-based approaches [78–80]. Our measured E2/T ratios in cord plasma were not significantly different between the control and intervention groups, and cord T levels were lower in female than male offspring. These data are in accordance with a meta-analysis on sex differences in human cord plasma T levels [81]. Importantly, we discovered lower E2/T ratio in female than male placentas due to higher T levels in female than male offspring placenta without differences in E2 levels between the sexes. Furthermore, we detected that these placental sex-differences in T levels and E2/T ratio were even enhanced upon the intervention. The placenta can act as barrier protecting mutually mother and fetus from an excess of different factors, including T [82, 83]. E2/T ratio is often used as an indirect marker for the activity of aromatase (*CYP19A1*) which is the key enzyme for the conversion of T to E2 or androstenedione to estrone [54, 84, 85]. Considering the data of Sathishkumar et al. [83], that aromatase mRNA and protein expression were lower in female than male placentas in normal human pregnancies, suggested reduced E2/T ratios due to lower E2 and/or increased T levels. Therefore, we hypothesized that the increased T levels in female placentas form the n-3 LCPUFA intervention group might be due to a downregulated expression of *CYP19A1*. Our gene and protein expression data however show a tendatively higher gene expression in male placentas, and tendatively decreased *CYP19A1* protein expression upon intervention. With regard to the impact of n-3 LCPUFA on circulating T levels in other human and animal studies on [55–59], it was also suggested that n-3 LCPUFAs influence T production in adult pig testis via their impact on PGE_2_ synthesis or their regulation of *PPAR*-mediated stearidogenesis [55]. Whether similar mechanisms are operating in the placenta needs detailed analysis in future research.

The observed altered placental T levels and E2/T ratio implied to be involved in the observed sex-specific gene expression which is in accordance with Clifton [9]. However, our analyses showed that for placental E2/T ratio or cord plasma T levels in total only two out of 7 genes with sex-specific regulations were significantly correlated suggesting that these factors might be involved only to a minor extent in the direct regulation of these genes. This is in concordance with Mao et al. [68] showing at least in mice that different maternal nutrition during pregnancy led to sexual dimorphism in the placental transcriptome at a time-point where the contribution of sex hormones seems unlikely.

Our bioinformatical approach to evaluate the potential that sex steroid hormones are implicated directly in the observed sex-specific gene regulation via their hormone receptors and putative hormone-responsive elements in the vicinity of identified target genes, surprisingly revealed only estrogen receptor alpha response elements for some of the genes, but no androgen receptor elements. Interestingly, with regard to the 'Wnt signaling' genes *DVL1*, *FZD7*, *LRP6*, and *SFRP1* at least one estrogen receptor alpha response element was annotated. The same was observed for *LPL*, and for *CYP19A1* three estrogen receptor alpha response elements were annotated.

Altogether, these observations raise several questions regarding the underlying mechanisms of sexually dimorphic gene expression and respective potential involvement of sex steroid hormones, such as (i) whether changes in E2/T ratios can modulate estrogen-driven gene regulation, (ii) if the analyzed placental hormones and their receptors mediated their action in addition to direct binding to cognate DNA sequences by indirect mechanisms such as recruitment to DNA through other transcription factors (tethering), or (iii) if affected gene transcription was the result of signaling cascades by non-genomic mechanisms of hormone action. Thus, more specific and detailed research is needed to find molecular insights into how sexually dimorphic gene expression in human somatic tissues, and in clinical traits such as obesity, artherosclerosis, and cardiovascular diseases, is regulated, and its underlying mechanistic relations to endo- and intracrinology.

## Conclusions

Altogether, we revealed sexually dimorphic gene expression in the human term placenta and discovered for the first time that maternal dietary n-3 LCPUFA intervention during pregnancy has a more pronounced impact on female than male placental gene expression, especially in the biological process of cell cycle. Our data underscore the importance to perform sex-specific analysis for the detection of sex-biased differences which will contribute to a better understanding of embryonic/extraembryonic development, biomedical research, and fetal and metabolic programming of offspring health and disease risk. Despite the absence of impact on birth weight or body composition upon n-3 LCPUFA intervention, the observed n-3 LCPUFA-responsive changes in placental gene expression pattern may contribute to programming effects on adipose tissues which may manifest later in life or on other tissues and physiological processes that have not been yet explored. Moreover, it will be interesting to investigate whether the observed n-3 LCPUFA-responsive gene expression changes in the placenta mirror gene activities and underlying epigenetic mechanisms which might also operate in maternal or other offspring tissues.

### Availability of supporting data

Gene expression data used for this study are available at NCBI’s Gene Expression Omnibus, GEO Series accession number GSE53291 (http://www.ncbi.nlm.nih.gov/geo/query/acc.cgi?acc=GSE53291). All other data sets supporting the results of this article are included within the article and its additional file.

### Abbreviations of gene names

*ACTB*, β actin; *ANAPC4*, anaphase promoting complex subunit 4; *B2M*, β-2-microglobulin; *C8orf59*, chromosome 8 open reading frame 59; *CDK1*, cyclin-dependent kinase 1; *CDK6*, cyclin-dependent kinase 6; *CKS2*, *CDC28* protein kinase regulatory subunit 2; *CYP19A1*, aromatase; *DDX3Y*, DEAD (Asp-Glu-Ala-Asp) box helicase 3, Y-linked; *DKK1*, dickkopf Wnt signaling pathway inhibitor 1; *DVL1*, dishevelled segment polarity protein 1; *EIF1AY*, eukaryotic translation initiation factor 1A, Y-linked; *EPS8L2*, *LIMCH EPS8*-like 2; *FZD7*, frizzled family receptor 7; *HDAC5*, histone deacetylase 5; *HDHD1A*, haloacid dehalogenase-like hydrolase domain containing 1; *KDM5D*, lysine (K)-specific demethylase 5D; *KDM6A*, lysine (K)-specific demethylase 6A; *LIMCH1*, LIM and calponin homology domains 1; *LPL*, lipoprotein lipase; *LRP6*, low density lipoprotein receptor-related protein 6; *MAD2L1*, *MAD2* mitotic arrest deficient-like 1; *PCNA*, proliferating cell nuclear antigen; *POLR2a*, polymerase (RNA) II (DNA directed) polypeptide A; *PPAR*, peroxisome proliferator-activated receptor; *RPS4Y1*, ribosomal protein S4, Y-linked 1; *SFRP1*, secreted frizzled-related protein 1; *SSB*, Sjogren syndrome antigen B (autoantigen La); *STRA6*, stimulated by retinoic acid 6; *TGFB1*, transforming growth factor beta 1; *TOP1*, topoisomerase (DNA) I; *USP9Y*, ubiquitin specific peptidase 9, Y-linked; *UTY*, ubiquitously transcribed tetratricopeptide repeat containing, Y-linked; *ZFY*, zinc finger protein, Y-linked; *ZNF711*, zinc finger protein 711.

## Electronic supplementary material

Additional file 1: **Text S1.** Expression analysis of PPARβ/δ and PPARγ target genes and genes involved in lipid metabolism. **Table S1.** Maternal and offspring clinical characteristics of the INFAT subpopulation subjected to DNA microarray analysis. **Table S2.** Maternal and offspring clinical characteristics of the INFAT subpopulation subjected to validations. **Table S3.** Oligonucleotides used for RT-qPCR analyses. **Table S4.** Summary of genes with sex-specific expression identified independently by placental transcriptome analyses in control placentas of this study, and by Sood et al. [15] and Tsai et al. [37]. **Table S5.** Placental gene expression of classical LCPUFA-regulated genes (literature selected), involved in lipid metabolism analyzed in microarray data sets of this study. **Table S6.** Expression changes of known PPAR β/δ target genes (literature-selected) analyzed in microarray data sets of this study. **Table S7.** Expression changes of known as PPARγ target genes (literature-selected) analyzed in microarray data sets in this study. **Table S8.** Sex steroid hormone analysis in placental tissue and cord plasma. **Table S9.** Correlation analyses for placental mRNA levels of significantly regulated genes with corresponding offspring anthropometric measurements up to one year. (PDF 184 KB)

## Abbreviations

Con: Control group
Con-F: Female offspring in the control group
Con-M: Male offspring in the control group
DHA: Docosahexaenoic acid
E2: Estradiol-17beta
EPA: Eicosapentaenoic acid
INFAT: **I**mpact of **N**utritional **F**atty Acids during Pregnancy and Lactation on early Human **A**dipose **T**issue Development
LCPUFA: Long-chain polyunsaturated fatty acid
N3: N-3 LCPUFA intervention group
N3-F: Placentas of female offspring in the n-3 LCPUFA intervention group
N3-M: Placentas of male offspring in the n-3 LCPUFA intervention group
T: Testosterone.
