# Supplementary material for: Human placental transcriptome shows sexually dimorphic gene expression and responsiveness to maternal dietary n-3 long-chain polyunsaturated fatty acid intervention during pregnancy
Source: BMC Genomics. 2014 Oct 27;15(1):941. doi: 10.1186/1471-2164-15-941 (PMC4232618; doi:10.1186/1471-2164-15-941)
Supplement: Supplementary file 1 — Additional file 1: Text S1. Expression analysis of PPARβ/δ and PPARγ target genes and genes involved in lipid metabolism. Table S1. Maternal and offspring clinical characteristics of the INFAT subpopulation subjected to DNA microarray analysis. Table S2. Maternal and offspring clinical characteristics of the INFAT subpopulation subjected to validations. Table S3. Oligonucleotides used for RT-qPCR analyses. Table S4. Summary of genes with sex-specific expression identified independently by placental transcriptome analyses in control placentas of this study, and by Sood et al. [15] and Tsai et al. [37]. Table S5. Placental gene expression of classical LCPUFA-regulated genes (literature selected), involved in lipid metabolism analyzed in microarray data sets of this study. Table S6. Expression changes of known PPAR β/δ target genes (literature-selected) analyzed in microarray data sets of this study. Table S7. Expression changes of known as PPARγ target genes (literature-selected) analyzed in microarray data sets in this study. Table S8. Sex steroid hormone analysis in placental tissue and cord plasma. Table S9. Correlation analyses for placental mRNA levels of significantly regulated genes with corresponding offspring anthropometric measurements up to one year. (PDF 184 KB) [file 12864_2014_6647_MOESM1_ESM.pdf]

Supplemental Text S1

Expression analysis of PPARβ/δ and PPARγ target genes and genes involved in lipid metabolism

Studies have often identified as dietary n-3 LCPUFAs supplementation effects expression changes for target genes of the transcription factors peroxisome proliferator-activated receptors (PPAR) and genes in lipid metabolism, especially in white adipose tissue and liver and corresponding cell culture models (86-93). Moreover, it has be shown that the activity for PPARα, β/δ and γ can be modulated by LCPUFAs and their metabolites in various tissues (89, 94, 95), and in gene knock-out mice the crucial roles of PPARγ and PPARβ/δ in placental development were demonstrated (96). In our study, placental microarray data sets were used to assess whether the n-3 LCPUFA intervention impacts the expression of similar genes or PPAR target genes which were extracted from the literature, such as 115

genes involved in lipid metabolism (89, 91, 93, 97, 98), 37 PPARβ/δ targets genes and 69 PPARγ target genes (94, 95). Due to absence of probes or very low hybridization intensities, 54 expressed genes, involved in lipid metabolism, qualified for comparison with our data sets. Thereof, only lipoprotein lipase (*LPL*) in data set (N3-F vs. Con-F) and medium-chain acyl-CoA dehydrogenase (*ACADM*) in data set (N3-M+N3-F vs. Con-M+F) showed significantly differential mRNA expression (see Additional file 6/Table S5).

With regard to PPARβ/δ and PPARγ-mediated gene expression, the genes for the transcription factors *PPARβ/δ* and *PPARγ* were not differentially expressed. For PPARβ/δ target genes, only Kruppel-like factor 11 (*KLF11*) showed significantly differential gene expression, whereas the genes for perilipin 2 (*PLIN2 / ADRP*) and hydroxysteroid (11-beta) dehydrogenase 1 (*HSD11B1*), known as PPAR β/δ target genes in trophoblast cells (99, 100), were not regulated (see Additional file 7/Table S6). Moreover, for PPARγ target genes, only *IGFBP1* and *LPL* showed significantly differential mRNA expression (see Additional file 8/Table S7). Comparing placentas of the intervention group to control group, *LPL* mRNA was higher expressed in female than male placentas upon intervention, whereas *IGFBP1* mRNA levels were significantly higher in male placentas upon intervention. PPARγ target genes mucin 1, cell surface

associated (*MUC1*), *ADRP*, *SLC27A1* (*FATP1*) and *SLC27A4* (*FATP4*), which have been detected in placental tissues (101), did not show significant regulations in the microarray data sets upon the intervention.

Supplemental References

86. Al Hasani H, Joost HG: **Nutrition-/diet-induced changes in gene expression in white adipose tissue.** *Best Pract Res Clin Endocrinol Metab*. 2005, **19**:589-603.
87. Deckelbaum RJ, Worgall TS, Seo T: **n-3 fatty acids and gene expression.** *Am J Clin Nutr*. 2006, **83**:1520S-1525S.
88. Flachs P, Rossmeisl M, Bryhn M, Kopecky J. **Cellular and molecular effects of n-3 polyunsaturated fatty acids on adipose tissue biology and metabolism.** *Clin Sci (Lond)*. 2009, **116**:1-16.
89. Jump DB, Botolin D, Wang Y, Xu J, Demeure O, Christian B: **Docosahexaenoic acid (DHA) and hepatic gene transcription.** *Chem Phys Lipids*. 2008, **153**:3-13.
90. Raclot T, Oudart H: **Selectivity of fatty acids on lipid metabolism and gene expression.** *Proc Nutr Soc*. 1999, **58**:633-646.
91. Sampath H, Ntambi JM: **Polyunsaturated fatty acid regulation of genes of lipid metabolism.** *Annu Rev Nutr*. 2005, **25**:317-340.
92. Simopoulos AP: **Human requirement for N-3 polyunsaturated fatty acids.** *Poult Sci*. 2000, **79**:961-970.
93. Vemuri M, Kelley DS 2007: **The effects of dietary fatty acids on lipid metabolism.** In: Chow CK (ed). Fatty acids in foods and their health implications.CRC Press Taylor and Francis Group, Boca Raton; FL:591-630
94. Adhikary T, Kaddatz K, Finkernagel F, Schonbauer A, Meissner W, Scharfe M, Jarek M, Blocker H, Muller-Brusselbach S, Muller R: **Genomewide analyses define different modes of transcriptional regulation by peroxisome proliferator-activated receptor-beta/delta (PPARbeta/delta).** *PLoS One*. 2011, **6**:e16344.
95. Keen HL, Halabi CM, Beyer AM, de Lange WJ, Liu X, Maeda N, Faraci FM, Casavant TL, Sigmund CD: **Bioinformatic analysis of gene sets regulated by ligand-activated and dominant-negative peroxisome proliferator-activated receptor gamma in mouse aorta.** *Arterioscler Thromb Vasc Biol*. 2010, **30**:518-525.
96. Barak Y, Sadovsky Y, Shalom-Barak T: **PPAR Signaling in Placental Development and Function.** *PPAR Res*. 2008, **2008**:142082.

97. Johnsen GM, Weedon-Fekjaer MS, Tobin KA, Staff AC, Duttaroy AK: **Long-chain polyunsaturated fatty acids stimulate cellular fatty acid uptake in human placental choriocarcinoma (BeWo) cells.** *Placenta*. 2009, **30**:1037-1044.

98. Oh DY, Talukdar S, Bae EJ, Imamura T, Morinaga H, Fan W, Li P, Lu WJ, Watkins SM, Olefsky JM: **GPR120 is an omega-3 fatty acid receptor mediating potent anti-inflammatory and insulin-sensitizing effects.** *Cell*. 2010, **142**:687-698.

99. Julan L, Guan H, van Beek JP, Yang K: **Peroxisome Proliferator-Activated Receptor +¦ Suppresses 11+¦-Hydroxysteroid Dehydrogenase Type 2 Gene Expression in Human Placental Trophoblast Cells.** *Endocrinology*. 2005, **146**:1482-1490.

100. Nadra K, Anghel SI, Joye E, Tan NS, Basu-Modak S, Trono D, Wahli W, Desvergne B: **Differentiation of trophoblast giant cells and their metabolic functions are dependent on peroxisome proliferator-activated receptor beta/delta.** *Mol Cell Biol*. 2006, **26**:3266-3281.

101. Schaiff WT, Barak Y, Sadovsky Y: **The pleiotropic function of PPAR gamma in the placenta.** *Mol Cell Endocrinol*. 2006, **249**:10-15.

Table S1: **Clinical characteristics and LCPUFA biomarker of mothers and offspring in the population for DNA microarray analysis.**

		Male offspring (M)		Female offspring (F)			P*	P#	P*#
		n	Mean ± SD / Median (IQR)	n	Mean ± SD / Median (IQR)	Con-M vs. Con-F	N3-M vs. N3-F	N3-F vs. Con-F	N3-M vs. Con-M
**Maternal characteristics**									
Maternal age (years)	**Con**	3	31.3 ± 2.5	4	30.0 ± 3.7		0.589	0.125	0.299
	**N3**	5	35.2 ± 5.2	4	28.8 ± 5.6				
Primiparae‡	**Con**	3	33.3% n: 1	4	0.0% n: 0	Fisher exact test			
	**N3**	5	40.0% n: 2	4	50.0% n: 2	1.000			
Weight before pregnancy (kg)	**Con**	3	66.7 ± 10.0	4	66.5 ± 5.4		0.804	0.320	0.303
	**N3**	5	61.0 ± 7.1	4	70.0 ± 10.9				
BMI before pregnancy (kg/m^2)†	**Con**	3	21.3 (20.7 - 23.5)	4	24.5 (21.9 - 26.2)		0.881	0.139	0.690
	**N3**	5	21.1 (20.4 - 23.6)	4	25.0 (20.9 - 27.7)				
Gestational weight gain[1] (kg)	**Con**	3	17.3 ± 0.6	4	15.5 ± 4.8		0.418	0.518	0.132
	**N3**	5	12.6 ± 4.0	4	17.0 ± 3.7				
**Offspring characteristics**									
Gestational age (d)	**Con**	3	283.0 ± 6.6	4	283.5 ± 3.4		0.717	0.493	0.363
	**N3**	5	284.2 ± 2.7	4	280.8 ± 4.2				
Placental weight (g)†	**Con**	3	515.3 ± 91.1	4	616.5 ± 20.6		0.063	0.407	0.067
	**N3**	5	510.2 ± 103.6	4	485.0 ± 35.5				
Birth weight (g)	**Con**	3	3490.0 ± 395.1	4	3782.6 ± 185.5		0.450	0.636	0.120
	**N3**	5	3611.0 ± 159.7	4	3450.0 ± 333.4				
Weight 1 year (g)	**Con**	3	102577 ± 1110.4	4	9522.5 ± 927.8		0.768	0.286	0.997
	**N3**	5	100560 ± 1846.2	4	9327.5 ± 639.9				
Birth weight percentile	**Con**	3	36.3 ± 22.6	4	70.0 ± 17.2		0.455	0.115	0.153
	**N3**	5	44.2 ± 16.3	4	46.0 ± 26.5				
Birth height (cm)	**Con**	3	51.7 ± 2.1	4	51.5 ± 0.6		0.386	0.851	0.992
	**N3**	5	52.4 ± 1.5	4	52.3 ± 2.1				
Birth head circumference (cm)	**Con**	3	35.0 ± 1.0	4	35.3 ± 0.5		0.884	0.561	0.204
	**N3**	5	35.4 ± 0.7	4	34.8 ± 0.5				
Birth weight / length (g/cm)	**Con**	3	67.7 ± 8.8	4	73.4 ± 3.6		0.292	0.605	0.146
	**N3**	5	68.9 ± 2.7	4	66.1 ± 6.6				
Birth weight / placenta weight	**Con**	3	6.8 ± 0.6	4	6.1 ± 0.3		0.207	0.452	0.668
	**N3**	5	7.3 ± 1.7	4	7.2 ± 1.0				
**LCPUFA biomarker**									
n6/n3 LCPUFA ratio[2] RBC mat. P_15	**Con**	3	2.73 (2.16-2.98)	4	2.79 (2.26-3.35)		0.594	0.363	0.530
	**N3**	5	1.85 (1.80-5.24)	4	2.79 (2.14-4.07)				
n6/n3 LCPUFA ratio[2] RBC mat. P_32	**Con**	3	2.02 (1.80-2.62)	4	2.77 (2.32-7.61)		**0.019**	0.173	0.940
	**N3**	5	1.32 (1.01-2.03)	4	1.47 (1.35-4.40)	0.330	0.319	0.068	0.091
n-6/n-3 LCPUFA ratio[2] CB RBCs†	**Con**	3	4.54 (3.86-4.79)	3	5.54 (5.39-8.07)		**0.003**	**0.003**	0.533
	**N3**	5	2.50 (2.30-3.04)	4	4.37 (3.18-9.11)	0.060	**0.004**	0.058	**0.009**

Data are presented as n, mean ± SD or percentage for each of the four analysis groups (Con-M, Con-F, n3-M, n3-F). For quantitative variables the corresponding P values are calculated with two-way ANOVA (P, P#, P*#). †Not normal distributed variables or variables that violated homoscedascity were presented as median (interquartile range IQR = 25th-75th percentile). Their corresponding P values were calculated with two-way ANOVA on ranks (P*, P#, P*#). In case of a significant two-way ANOVA P value, post-hoc tests (grey shaded) were conducted with Holm-Sidak test to adjust the significance level (Con-M vs. Con-F, n3-M vs. n3-F, n3-F vs. Con-F and n3-M vs. Con-M). ‡ For qualitative variables the Fisher exact test was used. P values < 0.05 were considered as significant and marked bold; P* < 0.05 significant difference between the n-3 LCPUFA intervention and the control group, effect of the intervention, P# < 0.05 significant difference between male and female placentas, effect of offspring sex; P*# < 0.05 significant interaction between sex and the intervention. [1]last measured value at booking minus self-reported weight before pregnancy, [2]n-6/n-3 LCPUFA ratio: (C20:2n-6 + C20:3n-6 + C20:4n-6 +C22:2n-6 + C22:4n-6 +C22:5n-6) / (C20:3n-3 + C20:4n-3 + C20:5n-3 + C22:3n-3 + C22:5n-3 + C22:6n-3); mat., maternal; CB, cord blood.*

Table S2: **Clinical characteristics and LCPUFA biomarker of mothers and offspring in the subpopulation**

		Male offspring (M)		Female offspring (F)			P*	P#	P*#
		n	Mean ± SD / Median (IQR)	n	Mean ± SD / Median (IQR)	Con-M vs. Con-F	N3-M vs. N3-F	N3-F vs. Con-F	N3-M vs. Con-M
**Maternal characteristics**									
Maternal age (years)	**Con**	9	33.6 ± 3.4	11	32.0 ± 4.3		0.446	0.257	0.948
	**N3**	11	32.6 ± 5.1	10	30.8 ± 5.1				
Primiparae‡	**Con**	9	44.4% n 4	11	45.5% n 5	Chi-Square test			
	**N3**	11	54.5% n 6	10	60.0% n 6	0.880			
Weight before pregnancy (kg)	**Con**	9	60.9 ± 8.1	11	63.6 ± 8.2		0.941	0.061	0.415
	**N3**	11	59.1 ± 6.4	10	65.7 ± 7.9				
BMI before pregnancy (kg/m^2)†	**Con**	9	21.0 (19.2-24.0)	11	22.7 (20.9-25.5)		0.652	0.080	0.962
	**N3**	11	20.8 (20.1-22.3)	10	21.9 (20.7-24.0)				
Gestational weight gain[1] (kg)	**Con**	9	16.1 ± 3.8	11	16.6 ± 6.5		0.641	0.506	0.715
	**N3**	11	14.9 ± 3.7	10	16.4 ± 3.5				
**Offspring characteristics**									
Gestational age (d)	**Con**	9	279.4 ± 8.7	11	282.6 ± 4.8		0.811	0.649	0.309
	**N3**	11	281.1 ± 5.4	10	279.9 ± 7.5				
Placental weight (g)	**Con**	9	551.0 ± 94.4	11	552.2 ± 70.0		0.469	0.859	0.896
	**N3**	11	529.0 ± 99.9	10	536.9 ± 51.8				
Birth weight (g)	**Con**	9	3487.2 ± 280.8	11	3544.6 ± 298.0		0.993	0.922	0.577
	**N3**	11	3536.8 ± 167.8	10	3496.5 ± 338.0				
Weight 1 year (g)	**Con**	8	9636.3 ± 1073.7	11	9058.2 ± 922.4		0.365	0.184	0.745
	**N3**	11	9839.1 ± 1259.4	10	9486.0 ± 1020.7				
Birth weight percentile†	**Con**	9	48.0 (29.5-54.0)	11	54.0 (28.0-80.0)		0.857	0.210	0.900
	**N3**	11	41.0 (32.0-50.0)	10	57.5 (32.8-82.3)				
Birth height (cm)	**Con**	9	52.2 ± 1.6	11	51.4 ± 1.4		0.495	0.469	0.350
	**N3**	11	52.1 ± 1.7	10	52.2 ± 1.8				
Birth head circumference (cm)	**Con**	9	35.6 ± 1.1	11	35.0 ± 0.8		0.312	0.064	0.914
	**N3**	11	35.3 ± 0.9	10	34.7 ± 1.1				
Birth weight / length (g/cm)	**Con**	9	66.9 ± 6.2	11	69.0 ± 5.3		0.749	0.711	0.330
	**N3**	11	67.9 ± 2.5	10	66.9 ± 5.6				
Birth weight / placenta weight†	**Con**	9	6.4 (6.0-6.9)	11	6.5 (5.9-6.9)		0.540	0.713	0.610
	**N3**	11	6.9 (5.5-7.6)	10	6.6 (5.9-7.0)				
**LCPUFA biomarker**									
n6/n3 LCPUFA ratio[2] RBC mat. P_15	**Con**	9	2.64 (2.33-3.04)	11	2.45 (2.26-3.13)		0.276	0.301	0.136
	**N3**	11	2.14 (1.84-2.67)	10	2.74 (2.33-2.93)				
n6/n3 LCPUFA ratio[2] RBC mat. P_32	**Con**	9	2.32 (1.91-2.69)	11	2.86 (2.20-4.64)		**< 0.001**	0.160	0.650
	**N3**	11	1.32 (1.07-1.99)	10	1.46 (1.30-1.74)	0.195	0.488	**<0.001**	**0.001**
n-6/n-3 LCPUFA ratio[2] CB RBCs†	**Con**	8	4.90 (4.03-6.22)	9	5.39 (4.07-7.39)		**0.005**	0.106	0.291
	**N3**	9	2.62 (2.45-3.59)	8	3.88 (3.18-5.16)	0.680	0.062	0.172	**0.007**

Data are presented as n, mean ± SD or percentage for each of the four analysis groups (Con-M, Con-F, n3-M, n3-F). For quantitative variables the corresponding P values are calculated with two-way ANOVA (P, P#, P*#). †Not normal distributed variables or variables that violated homoscedascity were presented as median (interquartile range IQR = 25th-75th percentile). Their corresponding P values were calculated with two-way ANOVA on ranks (P*, P#, P*#). In case of a significant two-way ANOVA P value, post-hoc tests (grey shaded) were conducted with Holm-Sidak test to adjust the significance level (Con-M vs. Con-F, n3-M vs. n3-F, n3-F vs. Con-F and n3-M vs. Con-M). ‡ For qualitative variables the Chi-Square test was used. P values < 0.05 were considered as significant and marked bold; P* < 0.05 significant difference between the n-3 LCPUFA intervention and the control group, effect of the intervention, P# < 0.05 significant difference between male and female placentas, effect of offspring sex; P*# < 0.05 significant interaction between sex and the intervention. [1]last measured value at booking minus self-reported weight before pregnancy, [2]n-6/n-3 LCPUFA ratio: (C20:2n-6 + C20:3n-6 + C20:4n-6 +C22:2n-6 + C22:4n-6 +C22:5n-6) / (C20:3n-3 + C20:4n-3 + C20:5n-3 + C22:3n-3 + C22:5n-3 + C22:6n-3); mat., maternal; CB, cord blood.*

Table S3: Oligonucleotides used for RT-qPCR analyses.

Gene name		Sequence (5´-3´)	Gene accession no.	Primer location on exon	Length (bp)	Temp (°C)
hACTB	for	CCTGGAGAAGAGCTACGAGCTG	NM_001101.3	3 - 4	108	60
	rev	GACTCCATGCCCAGGAAGGAAGG				
hPolR2a	for	CTTGTGTGATACCATGACCTGTCGTG	NM_000937.4	25 - 26	115	60
	rev	GCACGTCCACCGTTTCCTCAAAGG				
hB2M	for	GGACTGGTCTTTCTATCTCTTGTAC	NM_004048.2	2 - 3	120	60
	rev	TCTCGATCCCACTTAACTATCTTGG				
hTOP1	for	CTTCATCGACAAGCTTGCTCTG	NM_003286.2	14 - 15	98	60
	rev	TGATGTGCTCCACACGAAGTGA				
HDAC5	for	CCTACAGCAGAAGTTGAACGTGG	NM_000660.4	20 - 21	139	60
	rev	GAAGTTCCCGTTGTCATAGCGATG				
TGFB1	for	TGGTTGAGCCGTGGAGGGG	NM_005474.4	3 - 4	98	63
	rev	AACCCGTTGATGTCCACTTGCA				
DKK1	for	ACA ACT ACC AGC CGT ACC CGT	NM_012242.2	1 - 2	116	60
	rev	CTT CCT GCA GGC GAG ACA GAT				
DVL1	for	CAGCAGAGTGAAGGGAGCAAA	NM_004421.2	14 - 15	117	60
	rev	TGTGATCCGATTCACTGCCACT				
LRP6	for	GGATCTCTTCCAGGAATGTCTCG	NM_002336.2	20 - 21	150	60
	rev	TGCAGGGAAGTAAGTGCCTTTGG				
LPL	for	AAACCCATACCAATCAGGCCTTTG	NM_000237	7 - 8	93	63
	rev	TTGTGGAAACTTCAGGCAGAGTGA				
CYP19A1	for	GAA GGT CCT GTG CTC GGG	X55983	1.1 - 2	296	60
	rev	CAG GCA CTG CCG ATC CC				
ANAPC4		QT00027153	NM_013367	3 - 4	70	55
CDK1		QT00042672	NM_001786	1- 3	133	55
CDK6		†QT00019985	NM_001259	5 - 7	82	55
MAD2L1		QT00094955	NM_002358	1 - 2	104	55
PCNA		†QT00024633	NM_002592	5 - 6	105	55
FZD7		QT01010919	NM_003507	only 1 Exon	64	55
SFRP1		QT00031927	NM_003012	2 - 3	91	55

for, forward primer; rev, reverse primer; † commercial primers were purchased from Qiagen (Hilden, Germany); § this gene consists only of one exon; Temp, annealing temperature.

Table S4: Summary of genes with sex-specific expression identified independently by placental transcriptome analyses in control placentas of this study, and by Sood et al [15] and Tsai et al [37].

Gene symbol	Gene description	Location on Chr	Con-M vs. Con-F FC	P
CD99L2	CD99 molecule-like 2	X	1.92	0.0461
EIF1AX	eukaryotic translation initiation factor 1A, X-linked	X	-1.67	0.0447
HDHD1A	haloacid dehalogenase-like hydrolase domain containing 1A	X	-1.81	0.0013
KDM6A	lysine (K)-specific demethylase 6A	X	-1.87	0.0071
DDX3Y	DEAD (Asp-Glu-Ala-Asp) box polypeptide 3, Y-linked	Y	Expressed above background only in male placentas (fluorescence intensity above 20)	
EIF1AY	eukaryotic translation initiation factor 1A, Y-linked	Y		
KDM5D	lysine (K)-specific demethylase 5D	Y		
RPS4Y1	ribosomal protein S4, Y-linked 1	Y		
USP9Y	ubiquitin specific peptidase 9, Y-linked	Y		
UTY	ubiquitously transcribed tetratricopeptide repeat gene, Y-linked	Y		
ZFY	zinc finger protein, Y-linked	Y		
LPL	lipoprotein lipase	8	5.60	2.4 E-05

Chr, chromosome; FC, fold change; Con-M, placentas of male offspring in the control group; Con-F, placentas of female offspring in the control group.

Table S5: Placental gene expression of classical LCPUFA-regulated genes (literature selected), involved in lipid metabolism, analyzed in microarray data sets of this study.

Table S5a<br>Gene symbol	Gene description	N3-M+F vs. Con-M+F FC	P	N3-M vs. Con-M FC	P	N3-F vs. Con-F FC	P
**Gene group for fatty acid lipases**							
LPL	lipoprotein lipase	-1.61	>0.05	-3.62	>0.05	2.27	0.038
LIPE§	lipase, hormone-sensitive	1.06	>0.05	-1.02	>0.05	1.11	>0.05
LIPC§	lipase, hepatic	-1.03	>0.05	-1.03	>0.05	1.00	>0.05
PNLIP§	pancreatic lipase	1.04	>0.05	-1.10	>0.05	1.09	>0.05
**Gene group for long-chain fatty acid receptors**							
GPR120§	omega-3 fatty acid receptor 1	-1.10	>0.05	-1.02	>0.05	-1.04	>0.05
FFAR1§	free fatty acid receptor 1	1.23	0.030	1.16	0.0452	1.07	>0.05
**Gene group for fatty acid transport**							
CD36	CD36 molecule (thrombospondin receptor)	1.26	>0.05	1.55	>0.05	-1.21	>0.05
FABP1§	fatty acid binding protein 1, liver	1.03	>0.05	1.05	>0.05	-1.04	>0.05
FABP2§	fatty acid binding protein 2, intestinal	1.09	>0.05	1.02	>0.05	1.00	>0.05
FABP3§	fatty acid binding protein 3, muscle and heart (mammary-derived growth inhibitor)	-1.07	>0.05	1.05	>0.05	-1.09	>0.05
FABP4	fatty acid binding protein 4, adipocyte	1.42	>0.05	1.20	>0.05	1.20	>0.05
FABP5	fatty acid binding protein 5 (psoriasis-associated)	not annotated					
FABP6§	fatty acid binding protein 6, ileal	1.07	>0.05	-1.02	>0.05	-1.01	>0.05
FABP7§	fatty acid binding protein 7, brain	1.04	>0.05	1.00	>0.05	1.03	>0.05
FABP9	fatty acid binding protein 9, testis	no probe sets on microarray					
GOT2§	glutamic-oxaloacetic transaminase 2, mitochondrial (aspartate aminotransferase 2)	-1.18	>0.05	1.15	>0.05	-1.11	>0.05
SLC27A1§	solute carrier family 27 (fatty acid transporter), member 1	-1.03	>0.05	-1.02	>0.05	1.01	>0.05
SLC27A2	solute carrier family 27 (fatty acid transporter), member 2	-1.58	>0.05	1.05	>0.05	-1.62	>0.05
SLC27A3	solute carrier family 27 (fatty acid transporter), member 3	1.06	>0.05	1.15	>0.05	-1.04	>0.05
SLC27A4§	solute carrier family 27 (fatty acid transporter), member 4	1.20	0.047	1.12	>0.05	1.09	>0.05
SLC27A5§	solute carrier family 27 (fatty acid transporter), member 5	-1.16	>0.05	-1.04	>0.05	-1.10	>0.05
SLC27A6	solute carrier family 27 (fatty acid transporter), member 6	-1.55	>0.05	-1.24	>0.05	-1.25	>0.05
**Gene group for acyl CoA synthases**							
ACSL1	acyl-CoA synthetase long-chain family member 1	-1.19	>0.05	-1.08	>0.05	-1.18	>0.05
ACSL3	acyl-CoA synthetase long-chain family member 3	no probe sets on microarray					
ACSL4	acyl-CoA synthetase long-chain family member 4	-1.48	>0.05	-1.66	>0.05	1.22	>0.05
ACSL5§	acyl-CoA synthetase long-chain family member 5	1.03	>0.05	-1.01	>0.05	1.02	>0.05
ACSL6	acyl-CoA synthetase long-chain family member 6	no probe sets on microarray					
**Gene group for acyl-CoA binding proteins (DBI)**							
DBI	diazepam binding inhibitor (GABA receptor modulator, acyl-CoA binding protein)	1.04	>0.05	1.22	>0.05	-1.13	>0.05
PECI	enoyl-CoA delta isomerase 2	-1.57	>0.05	-1.09	>0.05	-1.50	>0.05
ACBP3-7	acyl-CoA-binding protein 3-7	no probe sets on microarray					
**Gene group for TG biosynthesis**							
DGAT1§	diacylglycerol O-acyltransferase 1	1.15	>0.05	1.07	>0.05	1.10	>0.05
DGAT2§	diacylglycerol O-acyltransferase 2	1.28	>0.05	1.11	>0.05	1.15	>0.05
GPAM§	glycerol-3-phosphate acyltransferase, mitochondrial	-1.15	>0.05	1.10	>0.05	-1.19	>0.05
LPIN1	lipin 1	-1.38	>0.05	-1.64	>0.05	1.24	>0.05
LPIN2	lipin 2	1.10	>0.05	1.09	>0.05	1.06	>0.05
MOGAT1§	monoacylglycerol O-acyltransferase 1	1.07	>0.05	1.18	0.024	-1.06	>0.05
MOGAT2§	monoacylglycerol O-acyltransferase 2	1.48	>0.05	-1.03	>0.05	1.45	0.028

Table S5b

Gene symbol	Gene description	N3-M+F vs. Con-M+F		N3-M vs. Con-M		N3-F vs. Con-F	
		FC	P	FC	P	FC	P
**Gene group for ß-oxidation**							
ACAA2[§]	acetyl-CoA acyltransferase 2	1.19	>0.05	1.15	0.034	1.10	>0.05
ACAA1[§]	acetyl-CoA acyltransferase 1	-1.08	>0.05	-1.10	>0.05	-1.03	>0.05
ACADM	acyl-CoA dehydrogenase, C-4 to C-12 straight chain	-1.67	0.024	-1.22	>0.05	-1.39	>0.05
ACADS[§]	acyl-CoA dehydrogenase, C-2 to C-3 short chain	1.03	>0.05	1.01	>0.05	1.12	>0.05
ACAD9	acyl-CoA dehydrogenase family, member 9	1.01	>0.05	-1.22	>0.05	1.30	>0.05
ACAD11	acyl-CoA dehydrogenase family, member 11	-1.16	>0.05	-1.26	>0.05	1.05	>0.05
ACADVL	acyl-CoA dehydrogenase, very long chain	1.30	>0.05	-1.08	>0.05	1.35	>0.05
ACOX1	acyl-CoA oxidase 1, palmitoyl	-1.11	>0.05	-1.00	>0.05	-1.10	>0.05
CPT1A	carnitine palmitoyltransferase 1A (liver)	-1.36	>0.05	-1.05	>0.05	-1.29	>0.05
CPT1B	carnitine palmitoyltransferase 1B (muscle)	no probe sets on microarray					
CPT1C[§]	carnitine palmitoyltransferase 1C	1.00	>0.05	1.00	>0.05	-1.01	>0.05
CPT2	carnitine palmitoyltransferase 2	-1.10	>0.05	1.01	>0.05	-1.13	>0.05
ECH1	enoyl CoA hydratase 1, peroxisomal	-1.12	>0.05	-1.05	>0.05	-1.07	>0.05
ECHS1	enoyl CoA hydratase, short chain, 1, mitochondrial	-1.16	>0.05	1.16	>0.05	-1.28	0.022
HADHA	hydroxyacyl-CoA dehydrogenase/3-ketoacyl-CoA thiolase/enoyl-CoA hydratase (trifunctional protein), alpha subunit	-1.01	>0.05	-1.08	>0.05	1.10	>0.05
HADHB	hydroxyacyl-CoA dehydrogenase/3-ketoacyl-CoA thiolase/enoyl-CoA hydratase (trifunctional protein), beta subunit	1.01	>0.05	1.06	>0.05	-1.05	>0.05
NDUFAB1	NADH dehydrogenase (ubiquinone) 1, alpha/beta subcomplex, 1, 8kDa	-1.05	>0.05	1.03	>0.05	1.00	>0.05
SLC25A20[§]	solute carrier family 25 (carnitine/acylcarnitine translocase), member 20	1.14	>0.05	1.05	>0.05	-1.07	>0.05
**Gene group for ß oxidation of uneven fatty acids**							
PCCA	propionyl CoA carboxylase, alpha polypeptide	-1.02	>0.05	-1.08	>0.05	1.04	>0.05
PCCB	propionyl CoA carboxylase, beta polypeptide	1.04	>0.05	-1.02	>0.05	1.00	>0.05
MCEE	methylmalonyl CoA epimerase	1.05	>0.05	1.11	>0.05	-1.14	>0.05
**Gene group for ß oxidation in unsaturated fatty acids**							
EHHADH[§]	enoyl-CoA, hydratase/3-hydroxyacyl CoA dehydrogenase	1.10	>0.05	1.12	>0.05	1.06	>0.05
ACADL[§]	acyl-CoA dehydrogenase, long chain	1.00	>0.05	-1.15	>0.05	1.04	>0.05
**Gene group for biosynthesis of saturated fatty acids**							
ACACA	acetyl-CoA carboxylase alpha	1.20	>0.05	1.07	>0.05	1.18	>0.05
ACACB[§]	acetyl-CoA carboxylase beta	1.12	>0.05	1.04	>0.05	1.07	>0.05
FASN[§]	fatty acid synthase	1.29	>0.05	-1.18	>0.05	1.49	.0003
**Gene group for biosynthesis of unsaturated fatty acids**							
FADS6[§]	fatty acid desaturase domain family, member 6	-1.05	>0.05	-1.03	>0.05	-1.01	>0.05
FADS2[§]	fatty acid desaturase 2	-1.06	>0.05	1.12	>0.05	-1.12	>0.05
ELOVL1	ELOVL fatty acid elongase 1	1.00	>0.05	1.04	>0.05	-1.03	>0.05
ELOVL2[§]	ELOVL fatty acid elongase 2	-1.14	>0.05	1.03	>0.05	-1.22	>0.05
ELOVL3[§]	ELOVL fatty acid elongase 3	1.03	>0.05	-1.05	>0.05	1.06	>0.05
ELOVL4[§]	ELOVL fatty acid elongase 4	-1.04	>0.05	-1.03	>0.05	-1.17	>0.05
ELOVL5	ELOVL fatty acid elongase 5	-1.23	>0.05	-1.11	>0.05	1.01	>0.05
ELOVL6[§]	ELOVL fatty acid elongase 6	1.01	>0.05	-1.01	>0.05	-1.03	>0.05
ELOVL7[§]	ELOVL fatty acid elongase 7	-1.30	>0.05	-1.42	>0.05	1.04	>0.05
SCD1	stearoyl-CoA desaturase (delta-9-desaturase)	1.02	>0.05	-1.21	>0.05	1.14	>0.05
**Gene group for biosynthesis of eicosanoides**							
PLA2G4A[§]	phospholipase A2, group IVA (cytosolic, calcium-dependent)	-1.35	>0.05	-1.24	>0.05	-1.03	>0.05
PLA2G4D[§]	phospholipase A2, group IVD (cytosolic)	-1.18	>0.05	-1.14	>0.05	1.00	>0.05
PLA2G4E	phospholipase A2, group IVE	not annotated					
PLA2G4F[§]	phospholipase A2, group IVF	1.04	>0.05	1.10	>0.05	1.02	>0.05

Table S5c<br>Gene symbol	Gene description	N3-M+F vs. Con-M+F		N3-F vs. Con-F		N3-M vs. Con-M	
		FC	P	FC	P	FC	P
**Gene group for biosynthesis of eicosanoids continued**							
PTGS1§	prostaglandin-endoperoxide synthase 1 (prostaglandin G/H synthase and cyclooxygenase)	1.03	>0.05	1.16	>0.05	-1.01	>0.05
PTGS2[§]	prostaglandin-endoperoxide synthase 2 (prostaglandin G/H synthase and cyclooxygenase)	1.37	>0.05	-1.16	>0.05	1.47	>0.05
ALOX12[§]	arachidonate 12-lipoxygenase	1.01	>0.05	1.01	>0.05	1.03	>0.05
ALOX12B[§]	arachidonate 12-lipoxygenase, 12R type	-1.05	>0.05	1.05	>0.05	-1.06	>0.05
ALOX15[§]	arachidonate 15-lipoxygenase	-1.03	>0.05	-1.02	>0.05	-1.03	>0.05
ALOX15B[§]	arachidonate 15-lipoxygenase, type B	1.07	>0.05	1.05	>0.05	1.02	>0.05
ALOX5	arachidonate 5-lipoxygenase	-1.00	>0.05	1.02	>0.05	-1.03	>0.05
ALOX5AP	arachidonate 5-lipoxygenase-activating protein	-1.31	>0.05	-1.24	>0.05	-1.02	>0.05
ALOXE3[§]	arachidonate lipoxygenase 3	1.16	>0.05	-1.08	>0.05	1.12	>0.05
**Gene group for storage and release of PUFA in TG and PL**							
AGPAT1	1-acylglycerol-3-phosphate O-acyltransferase 1 (lysophosphatidic acid acyltransferase, alpha)	1.03	>0.05	1.05	>0.05	-1.08	>0.05
PPAP2A	phosphatidic acid phosphatase type 2A	-1.39	>0.05	-1.03	>0.05	-1.39	>0.05
PPAP2B	phosphatidic acid phosphatase type 2B	1.13	>0.05	1.09	>0.05	1.04	>0.05
PPAP2C[§]	phosphatidic acid phosphatase type 2C	1.06	>0.05	1.03	>0.05	1.08	>0.05
ACAT1	acetyl-CoA acetyltransferase 1	-1.10	>0.05	-1.10	>0.05	-1.04	>0.05
CAV1	caveolin 1, caveolae protein, 22kDa	-1.08	>0.05	1.05	>0.05	-1.17	>0.05
PLIN2	perilipin 2	1.76	>0.05	-1.08	>0.05	1.97	>0.05
PPARA[§]	peroxisome proliferator-activated receptor alpha	-1.00	>0.05	1.04	>0.05	-1.02	>0.05
PPARG	peroxisome proliferator-activated receptor gamma	-1.27	>0.05	1.03	>0.05	-1.21	>0.05
PPARGC1[§]A	peroxisome proliferator-activated receptor gamma, coactivator 1 alpha	1.25	0.046	1.06	>0.05	1.17	>0.05
RXRA	retinoid X receptor, alpha	-1.02	>0.05	-1.20	0.015	1.21	0.007
RXRB	retinoid X receptor, beta	1.11	>0.05	-1.08	>0.05	1.12	>0.05
RXRG[§]	retinoid X receptor, gamma	1.04	>0.05	-1.02	>0.05	1.04	>0.05
HNF4A	hepatocyte nuclear factor 4, alpha	not annotated					
HNF4B	hepatocyte nuclear factor 4, beta	no probe sets on microarray					
HNF4G[§]	hepatocyte nuclear factor 4, gamma	1.18	>0.05	1.01	>0.05	1.13	>0.05
NR1H3	nuclear receptor subfamily 1, group H, member 3	1.04	>0.05	1.04	>0.05	1.04	>0.05
SREBF1[§]	sterol regulatory element binding transcription factor 1	1.32	>0.05	-1.23	>0.05	1.57	>0.05
SREBF2	sterol regulatory element binding transcription factor 2	no probe sets on microarray					
NFKB1	nuclear factor of kappa light polypeptide gene enhancer in B-cells 1	1.03	>0.05	1.02	>0.05	-1.03	>0.05

§ average intensity below 20; Con-M, placentas of male offspring in the control group; Con-F, placentas of female offspring in the control group; n3-M, placentas of male offspring in the n-3 LCPUFA intervention group; n3-F, placentas of female offspring in the n-3 LCPUFA intervention group; FC, fold change; *P*, P-value.

Table S6: Expression changes of known PPAR β / δ target genes (literature-selected) analyzed in microarray data sets of this study.

Table S6a

Gene symbol	Gene description	N3-M+F vs. Con-M+F		N3-M vs. Con-M		N3-F vs. Con-F	
		FC	P	FC	P	FC	P
ABCA1	ATP-binding cassette, sub-family A (ABC1), member 1	-1.02	>0.05	1.02	>0.05	-1.09	>0.05
ACAA2§	acetyl-CoA acyltransferase 2	1.19	>0.05	1.15	0.0343	1.10	>0.05
ACSL5§	acyl-CoA synthetase long-chain family member 5	1.03	>0.05	-1.01	>0.05	1.02	>0.05
PLIN2	perilipin 2	1.76	>0.05	-1.08	>0.05	1.97	>0.05
AHNAK	AHNAK nucleoprotein	-1.06	>0.05	-1.10	>0.05	-1.00	>0.05
AK3	adenylate kinase 3	-1.05	>0.05	-1.00	>0.05	-1.08	>0.05
ANGPTL4	angiopoietin-like 4	1.15	>0.05	-1.13	>0.05	1.37	>0.05
AP2A1§	adaptor-related protein complex 2, alpha 1 subunit	1.08	>0.05	-1.07	>0.05	1.15	>0.05
APH1B§	anterior pharynx defective 1 homolog B (C. elegans)	1.01	>0.05	-1.02	>0.05	-1.04	>0.05
BIRC3	baculoviral IAP repeat containing 3	no probe sets on microarray					
CDKN2C§	cyclin-dependent kinase inhibitor 2C (p18, inhibits CDK4)	-1.01	>0.05	1.09	>0.05	-1.06	>0.05
CNO	biogenesis of lysosomal organelles complex-1, subunit 4, cappuccino	1.30	>0.05	-1.07	>0.05	1.40	0.0068
CPT1a	carnitine palmitoyltransferase 1A (liver)	-1.36	>0.05	-1.05	>0.05	-1.29	>0.05
CPT2	carnitine palmitoyltransferase 2	-1.10	>0.05	1.01	>0.05	-1.13	>0.05
CSNK1G2§	casein kinase 1, gamma 2	-1.07	>0.05	-1.06	>0.05	1.00	>0.05
CTBS§	chitobiase, di-N-acetyl-	-1.13	>0.05	1.09	>0.05	-1.25	>0.05
CYB5D2§	cytochrome b5 domain containing 2	1.08	>0.05	1.07	>0.05	-1.03	>0.05
DCP1A	DCP1 decapping enzyme homolog A (S. cerevisiae)	-1.15	>0.05	-1.35	>0.05	1.20	0.0120
DIAPH1	diaphanous homolog 1 (Drosophila)	1.41	>0.05	-1.15	>0.05	1.48	0.0135
DOCK4	dedicator of cytokinesis 4	-1.65	>0.05	1.02	>0.05	-1.68	>0.05
ECH1	enoyl CoA hydratase 1, peroxisomal	-1.12	>0.05	-1.05	>0.05	-1.07	>0.05
ETFB	electron-transfer-flavoprotein, beta polypeptide	-1.10	>0.05	1.15	>0.05	-1.23	>0.05
ETFDH§	electron-transferring-flavoprotein dehydrogenase	1.07	>0.05	-1.02	>0.05	1.16	>0.05
EXOC6B§	exocyst complex component 6B	1.06	>0.05	-1.04	>0.05	1.08	>0.05
GPR180§	G protein-coupled receptor 180	-1.27	>0.05	-1.06	>0.05	-1.27	>0.05
GRAMD3	GRAM domain containing 3	1.30	>0.05	1.05	>0.05	1.26	>0.05
HMOX1	heme oxygenase (decycling) 1	-1.44	>0.05	-1.30	>0.05	-1.15	>0.05
HSD11B2	hydroxysteroid (11-beta) dehydrogenase 2	1.66	>0.05	1.24	>0.05	1.35	>0.05
HSDL2	hydroxysteroid dehydrogenase like 2	-1.77	>0.05	-1.25	>0.05	-1.54	>0.05
IFIT2	interferon-induced protein with tetratricopeptide repeats 2	-1.16	>0.05	-1.05	>0.05	-1.06	>0.05
IMPA2	inositol(myo)-1(or 4)-monophosphatase 2	1.49	>0.05	1.11	>0.05	1.32	>0.05
ISCA1	iron-sulfur cluster assembly 1 homolog (S. [illegible]	-1.55	>0.05	-1.10	>0.05	-1.41	0.0356
KLF10	Kruppel-like factor 10	-1.07	>0.05	1.17	>0.05	-1.22	0.0045
KLF11	Kruppel-like factor 11	-1.69	0.0398	-1.15	>0.05	-1.54	0.0144
MLYCD§	malonyl-CoA decarboxylase	1.11	>0.05	1.17	>0.05	-1.05	>0.05
MYO18A	myosin XVIIIA	not annotated					
NUDT13§	nudix (nucleoside diphosphate linked moiety X)-type motif 13	-1.01	>0.05	-1.02	>0.05	1.04	>0.05
NUDT9	nudix (nucleoside diphosphate linked moiety X)-type motif 9	-1.04	>0.05	-1.11	>0.05	1.07	>0.05
PDK4	pyruvate dehydrogenase kinase, isozyme 4	-1.25	>0.05	1.02	>0.05	-1.25	>0.05
PLIN1§	perilipin 1	1.09	>0.05	-1.01	>0.05	1.03	>0.05
PPARG	peroxisome proliferator-activated receptor gamma	-1.27	>0.05	1.03	>0.05	-1.21	>0.05
RUNX1§	runt-related transcription factor 1	1.01	>0.05	-1.07	>0.05	1.11	>0.05
SLC25A20§	solute carrier family 25 (carnitine/acylcarnitine translocase), member 20	1.14	>0.05	1.05	>0.05	-1.07	>0.05
SYTL3§	synaptotagmin-like 3	1.02	>0.05	1.06	>0.05	1.04	>0.05

**Table S6b**<br>**Gene symbol**	**Gene description**	**N3-M+ F vs. Con-M+F**		**N3-M vs. Con-M**		**N3-F vs. Con-F**	
		**FC**	**P**	**FC**	**P**	**FC**	**P**
TALDO1	transaldolase 1	1.05	>0.05	1.03	>0.05	-1.04	>0.05
TIMP4	TIMP metallopeptidase inhibitor 4	no probe sets on microarray					
TMEM135	transmembrane protein 135	no probe sets on microarray					
TNFRSF1A	tumor necrosis factor receptor superfamily, member 1A	-1.09	>0.05	-1.03	>0.05	-1.05	>0.05
TTC33	tetratricopeptide repeat domain 33	1.01	>0.05	1.02	>0.05	1.01	>0.05
UROS[§]	uroporphyrinogen III synthase	1.06	>0.05	1.01	>0.05	-1.05	>0.05
VAMP8	vesicle-associated membrane protein 8	1.13	>0.05	1.08	>0.05	1.02	>0.05
ZNF354A	zinc finger protein 354A	1.01	>0.05	-1.09	>0.05	1.04	>0.05
ZNF367[§]	zinc finger protein 367	-1.02	>0.05	1.24	0.0107	-1.26	0.0262

§ average intensity below 20; Con-M, placentas of male offspring in the control group; Con-F, placentas of female offspring in the control group; N3-M, placentas of male offspring in the n-3 LCPUFA intervention group; N3-F, placentas of female offspring in the n-3 LCPUFA intervention group; FC, fold change; *P*, P-value.

Table S7: Expression changes of known as PPARγ target genes (literature-selected) analyzed in microarray data sets in this study.

Gene symbol	Gene description	N3-M+F vs. Con-M+F		N3-M vs. Con-M		N3-F vs. Con-F	
		FC	P	FC	P	FC	P
ADIPOQ§	adiponectin, C1Q and collagen domain containing	-1.07	<0.05	-1.09	<0.05	1.04	<0.05
ANGPTL4	angiopoietin-like 4	1.15	<0.05	-1.13	<0.05	1.37	<0.05
APOE	apolipoprotein E	1.45	<0.05	-1.02	<0.05	1.30	<0.05
AQP7§	aquaporin 7	1.03	<0.05	1.08	<0.05	-1.02	<0.05
BCMO1§	beta-carotene 15,15'-monooxygenase 1	1.09	<0.05	1.11	<0.05	-1.01	<0.05
CAT	catalase	-1.39	<0.05	1.02	<0.05	-1.34	<0.05
CD36	CD36 molecule (thrombospondin receptor)	1.26	<0.05	1.55	<0.05	-1.21	<0.05
CIDEA§	cell death-inducing DFFA-like effector a	-1.07	<0.05	-1.01	<0.05	-1.06	<0.05
CPT1A	carnitine palmitoyltransferase 1A (liver)	-1.36	<0.05	-1.05	<0.05	-1.29	<0.05
DBI	diazepam binding inhibitor (GABA receptor modulator, acyl-CoA binding protein)	1.04	<0.05	1.22	<0.05	-1.13	<0.05
FABP4	fatty acid binding protein 4, adipocyte	1.42	<0.05	1.20	<0.05	1.20	<0.05
G0S2	G0/G1switch 2	1.18	<0.05	1.28	<0.05	-1.05	<0.05
GCK§	glucokinase (hexokinase 4)	1.06	<0.05	-1.01	<0.05	1.09	<0.05
IGFBP1*	insulin-like growth factor binding protein 1	1.60	<0.05	10.8	0.0433	-7.30	<0.05
IGFBP2	insulin-like growth factor binding protein 2, 36kDa	-1.38	<0.05	1.33	<0.05	-1.69	<0.05
IGFBP5	insulin-like growth factor binding protein 5	-1.18	<0.05	-1.25	<0.05	-1.01	<0.05
INSIG1	insulin induced gene 1	1.18	<0.05	-1.65	<0.05	2.00	<0.05
LPL*	lipoprotein lipase	-1.61	<0.05	-3.62	<0.05	2.27	0.0380
LRP1	ow density lipoprotein receptor-related protein 1	-1.18	<0.05	-1.03	<0.05	-1.09	<0.05
MC2R§	melanocortin 2 receptor (adrenocorticotropic hormone)	1.02	<0.05	1.03	<0.05	1.02	<0.05
MUC1	mucin 1, cell surface associated	1.20	<0.05	-1.34	<0.05	1.47	0.0460
NPHS1	nephrosis 1, congenital, Finnish type (nephrin)	no probe sets on microarray					
NR1D1§	nuclear receptor subfamily 1, group D, member 1	1.19	<0.05	1.13	<0.05	1.09	<0.05
NR1H3	nuclear receptor subfamily 1, group H, member 3	1.04	<0.05	1.04	<0.05	1.04	<0.05
PCK1§	phosphoenolpyruvate carboxykinase 1 (soluble)	-1.06	<0.05	-1.18	<0.05	1.22	0.0293
PLIN1§	perilipin 1	1.09	<0.05	-1.01	<0.05	1.03	<0.05
PLIN2	perilipin 2	1.76	<0.05	-1.08	<0.05	1.97	<0.05
PTGS2	prostaglandin-endoperoxide synthase 2 (prostaglandin G/H synthase and cyclooxygenase)	1.37	<0.05	-1.16	<0.05	1.47	<0.05
SCARB1	scavenger receptor class B, member 1	1.01	<0.05	-1.29	0.0426	1.36	<0.05
SCNN1G§	sodium channel, non-voltage-gated 1, gamma subunit	1.10	<0.05	-1.01	<0.05	1.11	<0.05
SERPINE 1	serpin peptidase inhibitor, clade E (nexin, plasminogen activator inhibitor type 1), member 1	-1.02	<0.05	1.14	<0.05	-1.17	<0.05
SLC1A2§	solute carrier family 1 (glial high affinity glutamate transporter), member 2	-1.09	<0.05	1.05	<0.05	-1.04	<0.05
SLC22A1§	solute carrier family 22 (organic cation transporter), member 1	-1.02	<0.05	1.01	<0.05	-1.06	<0.05
SLC27A1§	solute carrier family 27 (fatty acid transporter), member 1	-1.03	<0.05	-1.02	<0.05	1.01	<0.05
SLC27A4§	solute carrier family 27 (fatty acid transporter), member 4	1.20	0.0474	1.12	<0.05	1.09	<0.05
SLC2A2	solute carrier family 2 (facilitated glucose transporter), member 2	no probe sets on microarray					
SORBS1§	sorbin and SH3 domain containing 1	1.08	<0.05	1.02	<0.05	1.15	<0.05
TFF2§	trefoil factor 2	1.03	<0.05	-1.05	<0.05	1.16	0.0193
UCP1§	uncoupling protein 1 (mitochondrial, proton carrier)	1.11	<0.05	1.14	<0.05	-1.01	<0.05

§ average hybridization intensity below 20; * genes with significantly differential expressed genes; Con-M, placentas of male offspring in the control group; Con-F, placentas of female offspring in the control group; N3-M, placentas of male offspring in the n-3 LCPUFA intervention group; N3-F, placentas of female offspring in the N-3 LCPUFA intervention group; FC, fold change; *P*, P-value.

Table S8: Sex steroid hormone analysis in placental tissue and cord plasma.

Sex steroid hormones		Male			Female				***P****	***P#***	***P*#***
		n	median	(25th-75th percentile)	n	median	(25th-75th percentile)	**Con-M vs. Con-F**	**N3-M vs. N3-F**	**N3-F vs. Con-F**	**N3-M vs. Con-M**
CP free estradiol-17ß ng/ml†	**Con**	7	13.4	(11.4-38.0)	8	17.2	(13.1-32.0)		0.319	0.709	0.367
	**N3**	9	20.9	(18.7-24.3)	9	18.5	(11.7-26.0)				
CP conjugated estradiol-17ß ng/ml†	**Con**	7	272.5	(219.0-318.0)	8	247.8	(174.6-380.0)		0.188	0.778	0.190
	**N3**	9	158.0	(141.0-270.8)	9	281.5	(170.0-324.3)				
CP total free estrogene ng/ml	**Con**	7	26.9	(24.1-57.0)	8	42.6	(31.7-69.7)		0.290	0.495	0.210
	**N3**	9	43.1	(36.7-65.4)	9	49.1	(26.1-63.9)				
CP conjugated total estrogene ng/ml†	**Con**	7	132.8	(110.4-298.9)	8	111.0	(74.2-192.6)		0.083	0.847	0.094
	**N3**	9	79.3	(63.4-106.6)	9	120.0	(76.2-148.5)				
CP testosterone ng/ml†	**Con**	7	1.3	(1.0-1.3)	8	1.1	(0.8-1.3)		0.519	**0.049**	0.762
	**N3**	9	1.2	(1.0-1.4)	9	0.9	(0.8-1.1)	0.246	0.090	0.496	0.811
CP progesterone ng/ml†	**Con**	7	435.0	(358.0-803.0)	8	713.5	(367.0-857.5)		0.222	0.965	0.232
	**N3**	9	717.0	(481.0-1154.5)	9	629.0	(439.0-799.0)				
CP free estradiol-17ß / testoster-one ratio†	**Con**	7	12.9	(9.5-28.5)	8	17.0	(13.9-27.0)		0.619	0.563	0.428
	**N3**	9	18.4	(16.3-21.9)	9	19.6	(13.7-25.2)				
Placental free estradiol-17ß ng/g†	**Con**	9	53.2	(40.1-86.1)	11	50.9	(42.7-70.3)		0.830	0.396	0.616
	**N3**	11	65.4	(35.4-82.4)	10	50.6	(25.5-74.8)				
Placental conju-gated estradiol-17ß ng/g†	**Con**	9	23.3	(16.0-26.4)	11	19.4	(17.5-31.7)		0.192	0.609	0.800
	**N3**	11	25.0	(19.8-34.8)	10	24.6	(19.9-40.6)				
Placental free total estrogene ng/g†	**Con**	9	190.4	(128.6-330.8)	11	218.8	(167.9-278.7)		0.633	0.953	0.491
	**N3**	11	278.3	(126.3-389.8)	10	234.0	(111.5-324.0)				
Placental conju-gated total estrogene ng/g†	**Con**	9	11.8	(8.7-15.2)	11	11.2	(10.0-15.0)		0.979	0.984	0.920
	**N3**	11	13.2	(7.9-14.4)	10	12.4	(8.0-15.2)				
Placental testosterone ng/g	**Con**	9	10.6	(7.9-16.6)	11	14.8	(10.2-20.1)		0.252	**0.008**	0.278
	**N3**	11	10.7	(9.7-14.4)	10	19.7	(16.6-22.6)	0.249	**0.008**	0.113	0.965
Placental proges-terone ng/g†	**Con**	9	2837.0	(1398.5-1398.5)	11	3074.0	(2668.0-3312.0)		0.926	0.452	0.400
	**N3**	11	3432.0	(2326.0-3980.0)	10	2239.5	(1413.3-3600.8)				
Placental free estradiol-17ß / testosterone ratio†	**Con**	9	5.0	(3.0-6.6)	11	4.2	(2.5-6.1)		0.398	**0.013**	**0.045**
	**N3**	11	5.7	(3.6-6.3)	10	2.1	(1.7-3.7)	0.719	**0.002**	**0.042**	0.401

Sex steroid hormone data are presented as median with interquartile range (IQR: 25th - 75th percentiles). Statistical significance was calculated by two-way ANOVA with Holm-Sidak *post-hoc* test. †Not normal distributed sex steroid hormone parameters were tested for statistical significance by two-way ANOVA on ranks with Holm-Sidak *post-hoc* test. *P*, P-value; #, significant effects for sex differences; *, significant effects for the n-3 LCPUFA treatment; * #, significant interactions. $P < 0.05$ are marked in bold. C, control group; I, intervention group; CP, cord plasma; Con-M; placentas of male offspring in the control group; Con-F; placentas of female offspring in the control group; N3-M, placentas of male offspring in the n-3 LCPUFA intervention group, N3-F, placentas of female offspring in the n-3 LCPUFA intervention group.

Table S9: Correlation analyses for placental mRNA levels of significantly regulated genes with corresponding offspring body composition measurements up to one year.

	LRP6	DVL1	PCNA	CDK6	HDAC5	TGFB1
			**Birth weight**			
**Rs**	0.06	-0.02	**0.31**	**0.33**	0.06	-0.15
P	0.719	0.918	**0.049**	**0.042**	0.717	0.339
**N**	41	41	**41**	**38**	41	41
			**Birth weight percentiles**			
**Rs**	0.17	-0.04	**0.36**	**0.49**	0.04	-0.08
P	0.295	0.795	**0.023**	**0.002**	0.826	0.607
**N**	41	41	**41**	**38**	41	41
			**Ponderal index at birth**			
**Rs**	-0.06	0.03	0.16	-0.02	-0.05	-0.23
P	0.730	0.831	0.328	0.915	0.769	0.151
**N**	41	41	41	38	41	41
			**Birth weight to birth length ratio**			
**Rs**	0.06	0.01	**0.32**	0.26	0.04	-0.19
P	0.721	0.931	**0.039**	0.111	0.825	0.232
**N**	41	41	**41**	38	41	41
			**Birth weight to placental weight ratio**			
**Rs**	-0.12	0.16	0.08	0.26	0.28	0.18
P	0.471	0.325	0.626	0.111	0.079	0.250
**N**	41	41	41	38	41	41
			**Placental weight**			
**Rs**	0.13	-0.15	0.12	-0.09	-0.23	-0.30
P	0.402	0.366	0.462	0.595	0.151	0.055
**N**	41	41	41	38	41	41
			**Body weight at 1 year**			
**Rs**	0.03	**0.49**	0.15	0.22	**0.48**	0.20
P	0.834	**0.001**	0.350	0.193	**0.002**	0.210
**N**	40	**40**	40	37	**40**	40
			**Sum of four skin fold thicknesses (SFT) 3-5 days**			
**Rs**	-0.02	-0.31	0.29	0.31	-0.14	-0.07
P	0.924	0.056	0.074	0.067	0.387	0.661
**N**	39	39	39	36	39	39
			**Sum of four skin fold thicknesses (SFT) 1 year**			
**Rs**	0.13	0.07	0.04	0.04	-0.01	-0.16
P	0.422	0.665	0.832	0.802	0.956	0.336
**N**	40	40	40	37	40	40
			**Subcutaneous-to-preperitoneal fat mass ratio 6 weeks**			
**Rs**	0.20	0.01	-0.08	0.06	0.01	-0.09
P	0.273	0.961	0.669	0.758	0.971	0.619
**N**	32	32	32	29	32	32
			**Subcutaneous-to-preperitoneal fat mass ratio 1 year**			
**Rs**	0.32	-0.04	-0.14	0.25	-0.08	-0.07
P	0.057	0.800	0.416	0.149	0.643	0.673
**N**	37	37	37	34	37	37

Rs between 0.0 - 0.4 > 0.4 – 0.7 > and 0.7 – 1.0 were considered as weak, moderate and strong correlations, respectively. Positive and negative values indicate positive and inverse correlations. Correlation analysis was conducted independent of n-3 LCPUFA intervention status. *P* < 0.05 was considered as significant and is marked in bold. Rs, spearman-rho correlation coefficient; *P*, P-value for the respective correlation.
